# Combining computational modeling and experimental library screening to affinity‐mature VEEV‐neutralizing antibody F5

**DOI:** 10.1002/pro.70043

**Published:** 2025-01-22

**Authors:** Christopher A. Sumner, Jennifer L. Schwedler, Katherine Maia McCoy, Jack Holland, Valerie Duva, Daniel Gelperin, Valeria Busygina, Maxwell A. Stefan, Daniella V. Martinez, Miranda A. Juarros, Ashlee M. Phillips, Dina R. Weilhammer, Gevorg Grigoryan, Michael S. Kent, Brooke N. Harmon

**Affiliations:** ^1^ Department of Biotechnology and Bioengineering Sandia National Laboratories Livermore California USA; ^2^ Department of Molecular and Cell Biology Dartmouth College Hanover New Hampshire USA; ^3^ Department of Computer Science Dartmouth College Hanover New Hampshire USA; ^4^ Abcam Branford Connecticut USA; ^5^ Department of Molecular and Microbiology Sandia National Laboratories Albuquerque New Mexico USA; ^6^ Division of Biosciences and Biotechnology Lawrence Livermore National Laboratories Livermore California USA

**Keywords:** affinity‐maturation, anti‐viral, computational modeling, library screening, therapeutic antibodies

## Abstract

Engineered monoclonal antibodies have proven to be highly effective therapeutics in recent viral outbreaks. However, despite technical advancements, an ability to rapidly adapt or increase antibody affinity and by extension, therapeutic efficacy, has yet to be fully realized. We endeavored to stand‐up such a pipeline using molecular modeling combined with experimental library screening to increase the affinity of F5, a monoclonal antibody with potent neutralizing activity against Venezuelan Equine Encephalitis Virus (VEEV), to recombinant VEEV (IAB) E1E2 antigen. We modeled the F5/E1E2 binding interface and generated predictions for mutations to improve binding using a Rosetta‐based approach and dTERMen, an informatics approach. The modeling was complicated by the fact that a high‐resolution structure of F5 is not available and the H3 loop of F5 exceeds the length for which current modeling approaches can determine a unique structure. A subset of the predicted mutations from both methods were incorporated into a phage display library of scFvs. This library and a library generated by error‐prone PCR were screened for binding affinity to the recombinant antigen. Results from the screens identified favorable mutations which were incorporated into 12 human‐IgG1 variants. The best variant, containing eight mutations, improved KD from 0.63 nM (parental) to 0.01 nM. While this did not improve neutralization or therapeutic potency of F5 against IAB, it did increase cross‐reactivity to other closely related VEEV epizootic and enzootic strains, demonstrating the potential of this method to rapidly adapt existing therapeutics to emerging viral strains.

## 1 INTRODUCTION

In an increasingly interconnected world, the global risk of exposure to emerging pathogens makes the need for safe, potent therapeutic antibodies (Abs) ever more important for ensuring world health safety and security (Chames and Baty 2012; Hu and Nagata 2016). The adaptive immune system can be trained to naturally develop affinity matured Abs for a known pathogen through vaccination or natural exposure, but over the past 40 years, the field of Ab research has expanded and deepened our understanding of Ab structure, function, and engagement with the immune system, leading to our capacity to design and engineer novel, affinity‐matured, therapeutics against emerging infectious agents. Optimizing binding affinity can improve potency and specificity, thereby reducing off target binding and side effects. Furthermore, developing highly potent, effective Abs can lower the effective dose and reduce the production costs. The engineering process of affinity maturation poses an interesting challenge as it involves a large sequence space of proteins 20^*n*^, where *n* is the number of amino acids in a protein. Random mutagenesis of scFv‐based phage libraries typically results in 10^8^–10^10^ variants, and only a handful of mutations at critical complementarity‐determining region (CDR) positions typically impact Ab affinity. Thus, random mutagenesis for affinity maturation is a slow and inefficient process (Alfaleh et al. 2020; Frigotto et al. 2015).

In silico modeling holds promise to aid the process of affinity maturation through various structure‐based computational design (SBCD) methods. Access to a large and ever‐increasing protein database has facilitated the ability of computation to provide critical knowledge about fundamental interactions and the patterns of interacting amino acids in known structural motifs. We demonstrate with our modeling approach that computation can effectively screen mutants at a significantly higher rate than experimental approaches, thereby facilitating the requisite rapidity with which we must respond to new and emerging pathogens.

There are a variety of methods that may be utilized for SBCD of Ab–antigen (Ag) interactions (Norman et al. 2020). Generally, any de novo method must involve prediction of the individual structures, prediction or design of their binding conformation(s), and prediction of binding strength for variations in Ab or Ag sequence for a given conformation or combination of conformations. RosettaAntibodyDesign (Adolf‐Bryfogle et al. 2018) and OptCDR (Pantazes and Maranas 2010) both sample substructures of antibodies according to known canonical classes and graft them together. They then simulate mutating Ab residues followed by structural refinement with the mutations. Sequences are chosen with the best energies according to Rosetta energy and CHARMM energy respectively. OptMAVEn (Chowdhury et al. 2018) simulates Variable, Diversity, and Joining gene (VDJ) recombination; the process by which lymphocytes randomly rearrange these gene segments to produce a broad repertoire of B cell receptors—Abs—and T cell receptors from a database of Ab structures including heavy chain (HC), and light chains (LC, kappa, and lambda), then generates a docking ensemble and selects the structure with the best CHARMM energy. AbDesign aligns Abs and segments them according to points of maximum structural conservation, then grafts them together to generate many new backbones, followed by Rosetta sequence design (Lipsh‐Sokolik et al. 2020). Abs that already bind antigens, with known or predicted binding conformations, may also be redesigned to improve binding. For example, a given Ab variable region may be redesigned using a physics‐based energy function (Lippow et al. 2007), or Molecular Dynamics simulations combined with Monte Carlo sampling of different residues (Buratto et al. 2022) to target the same or a different epitope within an antigenic domain to achieve increased affinity and therapeutic potency. In this work we demonstrate our process of redesigning the anti‐VEEV Ab, F5, that is known to bind Venezuelan Equine Encephalitis Virus (VEEV) E1E2, and to improve upon its binding affinity and by extension efficacy.

VEEV is a New World alphavirus that can cause highly pathogenic neurological disease in humans and equines. The New World alphaviruses are considered potential biological weapons and are identified as Category B pathogens by the National Institute of Allergy and Infectious Diseases due to past weaponization, ease of producing large quantities of virus, and their highly infectious nature through aerosol exposure. There are currently no FDA‐approved vaccines or drugs to prevent or treat neurotropic infections caused by VEEV and similar encephalitic viruses (Cohen et al. 2016; Neira et al. 2007; Taylor et al. 2015; Wolfe et al. 2014), and thus we have an important opportunity to address these deficiencies by improving our ability to design and re‐design Ab therapeutics to meet the challenge of these and future neurovirulent agents.

F5 is a monoclonal Ab that is potently neutralizing for VEEV and binds to domain A of the VEEV E2 envelope glycoprotein (Hunt et al. 2010). F5 was isolated using a phage display Ab library created from human bone marrow donors known to have circulating Abs for VEEV. We and others have shown that F5 provides 90%–100% protection when given pre‐exposure (−24 h) and 70%–90% protection, when dosed +24 to +48 h post infection (hpi), in a lethal VEEV‐TC83 model, with some variation depending on the route of infection whether aerosol, intranasal or subcutaneous (AE, IN, or SQ) (Hunt et al. 2010; Schwedler et al. 2024). Similarly F5 protects with 90%–100% efficacy when dosed prophylactically (−24 h) by SQ injection, AE, or IN administration, but that efficacy drops precipitously to 30%–40% at +24hpi, for SQ and IN exposure, and to 0% at +48hpi by IN route with fully virulent VEEV IAB subtype Trinidad donkey (VEEV‐TrD) (Hunt et al. 2010). Interestingly, mice infected by aerosol challenge with VEEV‐TrD and treated with F5 at +24hpi developed central nervous system infections but little or no clinical signs of disease and showed an 80% survival rate (Cohen et al. 2016). Accordingly, while F5 may be a strong candidate for prophylactic treatment of VEEV infection, its efficacy as a therapeutic at +24 and +48hpi is lacking. Thus, it was our goal to optimize F5 using our computational and experimental affinity‐maturation pipeline to increase binding, and by extension enhance our therapeutic arsenal against encephalitic alphaviruses like VEEV.

In this work, computational modeling was combined with experimental library screening to improve the binding of the VEEV‐neutralizing Ab F5 to recombinant E1E2 spike protein trimer of VEEV‐TC83, an experimental live‐attenuated VEEV vaccine derivative of virulent VEEV‐TrD. The modeling was particularly challenging because a high‐resolution crystal structure was not available for F5, and the H3 loop of F5 is quite large (20 amino acids). Whereas the other CDR loops can be modeled from canonical structures, that is not the case for H3 due to its length (Jeliazkov et al. 2018; Weitzner and Gray 2017). This challenge was addressed using published experimental data for the binding affinity of F5 to VEEV subtypes TC‐83, IAB, IV, and V (Hunt et al. 2010). Nine F5 structures were docked to these various VEEV subtypes, and the complex that was most consistent with the published binding affinity data was selected for mutational analysis. Two modeling approaches were used to predict mutations to improve binding. The first approach was Sequence Tolerance (Smith and Kortemme 2010; Smith and Kortemme 2011) within the Rosetta Protein Modeling Suite, a Monte Carlo‐based tool for modeling structures and interactions of proteins. Benchmarking of Rosetta‐based approaches for modeling Ab structures and docking Ab structures to antigens has been reported elsewhere (Weitzner et al. 2017). The second approach was dTERMen, an informatics approach (Zhou et al. 2020). Subsets of the predictions from both approaches (29 mutations at 13 sites) were screened using a phage library displaying scFvs that incorporated nearly all combinations of the predicted mutations. Here, we report the modeling procedures and the results of the library screening, which led to the identification of Abs that displayed increased binding to the E1E2 heterodimer by more than 60‐fold, with significant improvement in the Kd (off rate) over the parental F5. Top candidate Abs displaying enhanced binding to the E1E2 heterodimer were tested for efficacy at binding and neutralization of virus *in vitro* as well as protection against lethal infection *i* *n vivo*.

## 2 RESULTS

### 2.1 *In silico* mutation analysis

**FIGURE 1:**
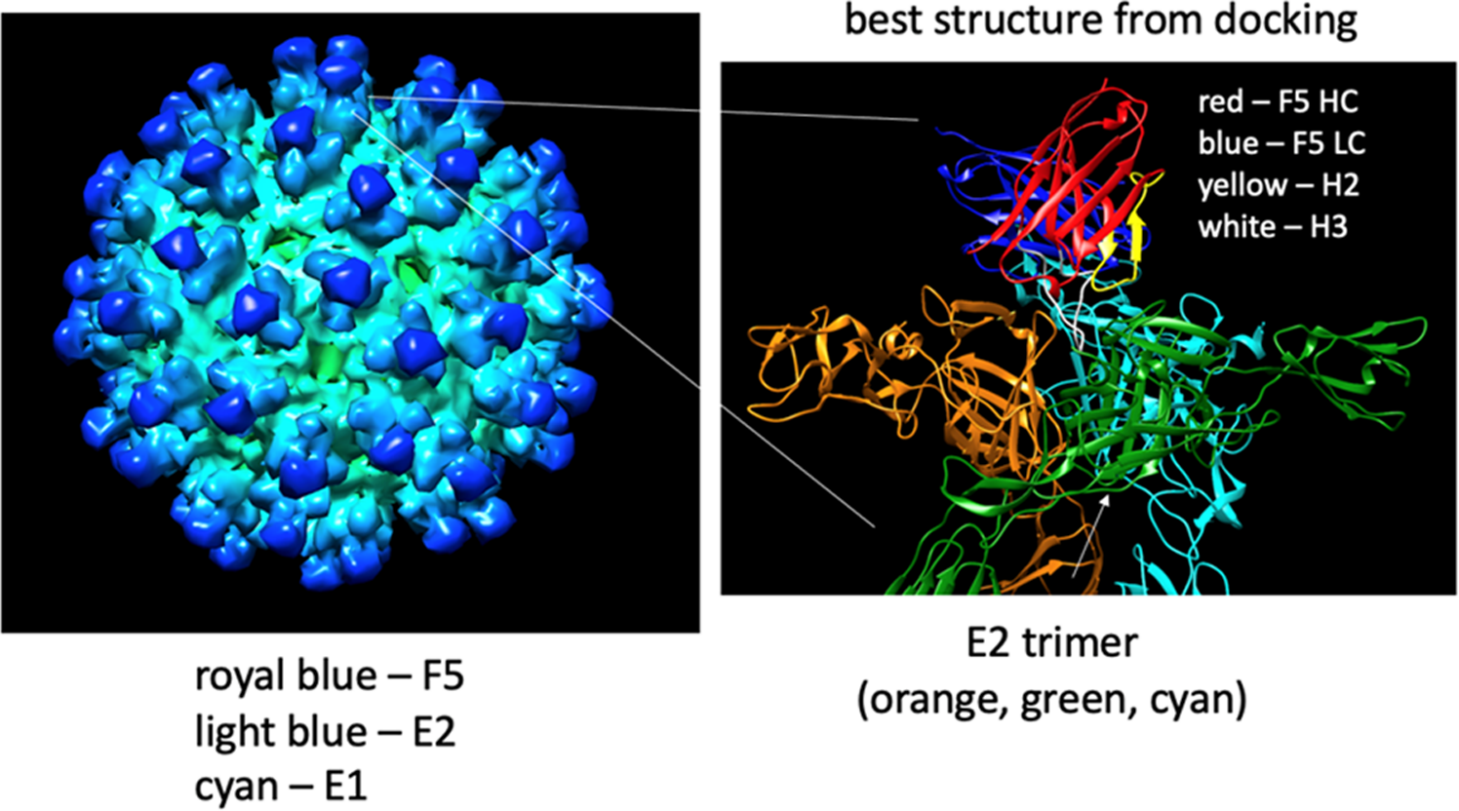
The structural model used for mutational analysis by Sequence Tolerance, FlexddG, and dTERMen.

Since the cryoEM structure EMD‐2645 indicates that F5 binds near the center of the three E2 monomers in the E1E2 trimer (Porta et al. 2014), and considering the large length of the H3 loop, it seems likely that the H3 loop interacts with and inserts into the open space in the center of the E1E2 trimer. Since a high‐resolution structure for F5 was not available, nine F5 models with varying H3 conformations were generated using RosettaAntibody, PIGS, and Swiss Model (Figure S1, Supporting Information).^1^ The H2 loop structure was very similar in eight of the nine models, whereas the structure of the H3 loop varied greatly among the nine models (Figure S1a). To select among these models the nine structures were used in docking trials against VEEV subtype IAB (TrD and TC‐83), IV, and V antigens using Rosetta protocols Docking2 and Snugdock. The results, shown in Figure S2, were evaluated based on the trends in relative binding affinity for each of the subtypes. A prior study reported the relative binding as TC‐83 ~ TrD > IV > V (Hunt et al. 2010). The RosettaAntbody1 structure gave results that were most in line with the experimentally observed trend. For that structure the docking results gave the binding affinity as TC‐83 ~ TrD > IV. The docking results for binding to subtype V were not consistent with the reported experimental trend, as no detectable binding was reported at 100 μg/mL for subtype V in the experimental study. Nevertheless, since the results were consistent with the trend for the other subtypes, this structure was selected and further refined in docking trials using the Relax function followed by two additional rounds of flexible docking using Snugdock on ROSIE (fast protocol). This final structure, shown in Figure 1, was used to generate predictions for mutations to improve binding.^2^

**FIGURE 2:**
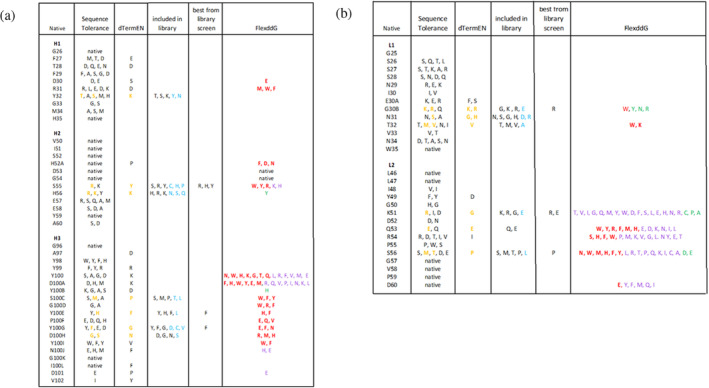
(a) Heavy chain and (b) light chain mutation predictions and results of library screening. The predicted mutations from Sequence Tolerance, dTERMen, and FlexddG mutations are shown along with mutations that were highly selected in the library screening. The mutations that were included in the library are shown in the fourth column. Mutations that were not predicted but were a consequence of including the predicted mutations are shown in cyan in the fourth column. The mutations predicted by Sequence Tolerance and dTERMen that were included in the library are shown in gold. For the FlexddG predictions, mutations predicted to be strongly favorable (>1.0 REU), moderately favorable (0.5–1.0 REU), and marginally favorable (0.2–0.5 REU) are shown in red, purple, and green, respectively.

The results of Sequence Tolerance Analysis for the CDR loops of the final refined F5‐VEEV structure are given in Figure S3. Considering the sites in direct contact with the antigen or only 1 site removed from direct contact, the analysis indicated that at 24 sites mutations were preferred or tolerated at comparable frequency to the parent residue. Among these, 19 mutations at 13 sites were included in the experimental library, indicated in gold in Figure 2.

dTERMen was also used to predict favorable mutations in the final refined F5‐VEEV structure. Two sets of predicted mutations, one based on pseudo‐energies alone and another based on pseudo‐energies with a light specificity cutoff (see section 5), are given in Figures S4 and S5. Considering the sites in direct contact with the antigen or only 1 site removed from direct contact, the results indicated favorable mutations at 25 sites. Among these, 15 mutations at 13 sites were included in the experimental library. Downselection was required due to constraints on the size of the experimental library. The mutations included in the experimental library are highlighted in gold in Figures S6 and 2. Of the 15 mutations, 10 were unique to dTERMen and 5 were in common with the predictions from Sequence Tolerance.

Subsequent to the experimental screening, FlexddG was performed for all sites at which mutations were included in the directed library. The results are given in Figure 2, where bold red indicates favorable ΔΔ*G* values greater than 1 Rosetta Energy Unit (REU), purple indicates favorable ΔΔ*G* values between 0.5 and 1.0 REU, and green indicates favorable ΔΔ*G* values between 0.2 and 0.5 REU.

### 2.2 Library screening

Two phage libraries were tested for binding to the recombinant VEEV (TC‐83) E1E2. Each library had a diversity of roughly 4 × 10^8^. One library was generated by randomly introducing mutations throughout the entire scFv, including the framework regions. The second library was generated by introducing specific mutations from the in silico modeling. Despite the large library size of 4 × 10^8^, not all predicted beneficial mutations could be screened. While larger libraries have been routinely made by many labs, including ours, we aimed here to generate a library with a smaller number of mutations that interrogates nearly all possible combinations of the mutations chosen for inclusion (Holland et al. 2013). A total of 19 mutations from Rosetta and 15 mutations from dTERMen (10 unique and 5 in common with Rosetta) at 13 sites were included in the directed library. These mutations were chosen based on the anticipated impact on binding as well as considerations from codon usage such as avoiding stop codons and minimizing the number of off‐target amino acids generated at each site of mutation. In addition to the desired mutations, an additional 21 mutations were present as a necessary consequence of the codons used to incorporate the desired mutations.

#### 2.2.1 Screening results: Directed library

**FIGURE 3:**
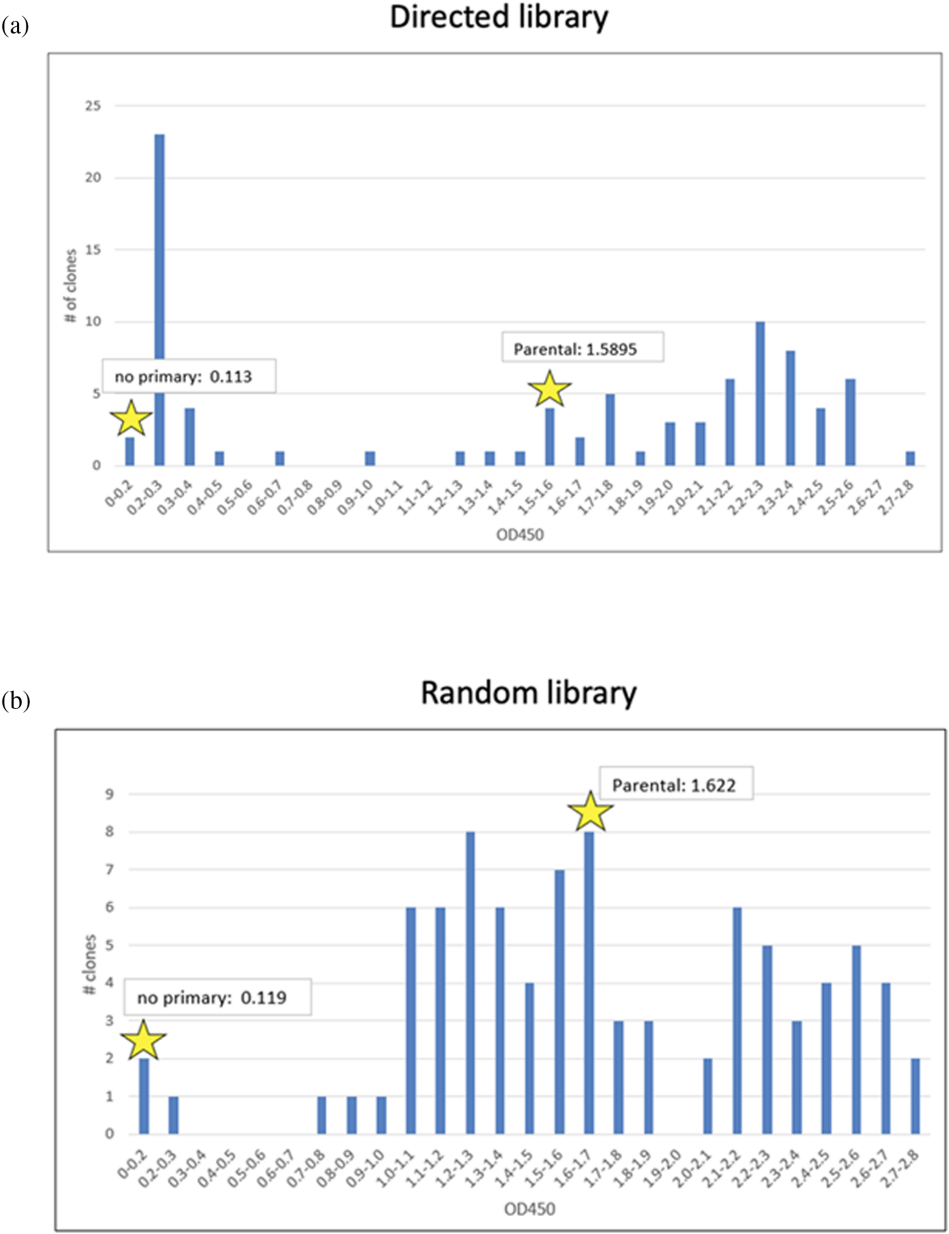
ELISA results for binding of clones to the E1E2 heterodimer for the (a) directed library and (b) the random library.

**FIGURE 4:**
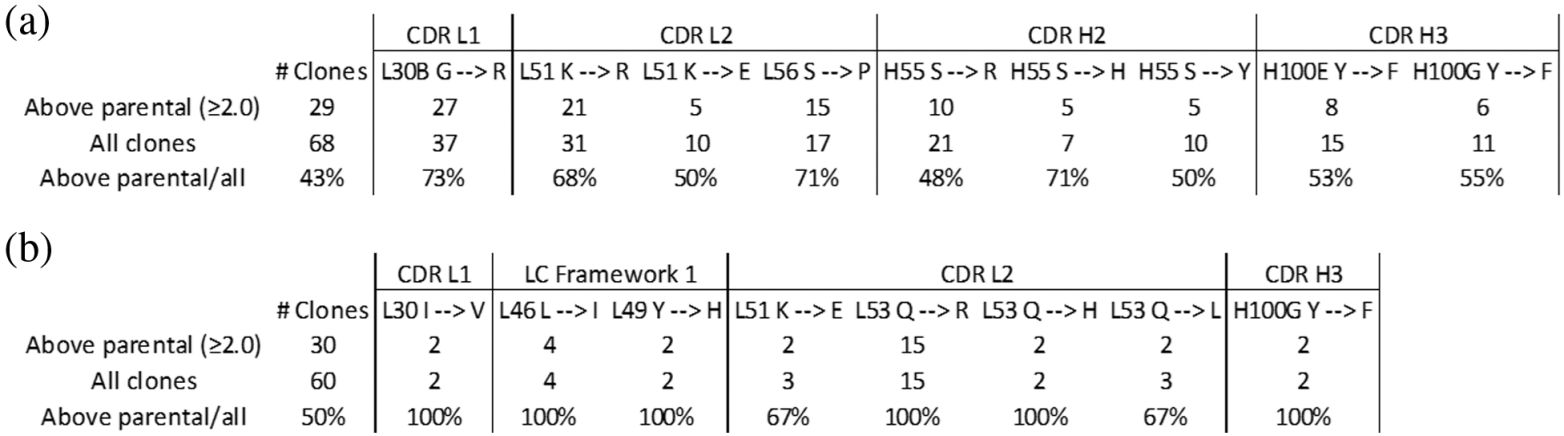
Summary of beneficial mutations for (a) directed library and (b) random library. The criteria used to assign a mutation as being clearly beneficial was that a mutation must have appeared in greater than three clones and roughly 50% or greater of all instances of that mutation must have occurred within the clones that gave higher binding than parental. The nine mutations shown in (a) meet those criteria whereas only two of the mutations in (b) meet those criteria.

After three rounds of affinity selection 68 clones were identified and sequenced. Based on the ELISA signal intensity shown in Figure 3, 29 unique clones were identified as having improved binding relative to the parental F5, 14 clones had comparable binding to the parental, and 23 clones had lower binding than the parental. Mutations were identified as beneficial, neutral, or detrimental based on their distribution among the clones in the three categories (improved, comparable, or lower binding relative to parental). The results are tabulated in Figures S7–S9. To assign a mutation as being clearly beneficial, we used the criteria that a mutation must have appeared in greater than three clones and roughly 50% or greater of all instances of that mutation must have occurred within the clones that gave higher binding than parental. We note that this latter criterion considers all mutations independently, even though other mutations in a clone will likely affect binding. By that criterion, nine mutations at six positions were identified as highly likely to be beneficial for improving binding (Figure 4). Three of the nine were uniquely predicted by the Rosetta method, three were uniquely predicted by dTERMen, one was predicted by both methods, and two were off‐target amino acids that were present because of the nucleotides required for the targeted amino acids. The latter demonstrates that there is value in simply identifying the interacting amino acids and focusing library diversity on those sites. Another important point is that simply combining all predicted mutations into a single construct did not lead to successful clones. For the 28 clones that had improved binding to the target, the number of mutations ranged from 2 to 6 with a mean of 3.8.

Regarding FlexddG, nine of the experimentally‐determined favorable mutations were correctly identified as favorable by FlexddG: four of these were predicted by FlexxddG to be strongly favorable (>1 REU), four were predicted to be mildly favorable (0.5–1.0 REU), and 1 was predicted to be weakly favorable (0.2–0.5 REU).

#### 2.2.2 Screening results: Random library

Based on the ELISA signal intensity shown in Figure 3, clones of the random library were also identified as having improved binding relative to the parental F5 although the number of clones with improved binding were fewer than for the directed library. Sequencing the clones indicated that, using the same criteria, two mutations were identified in the random library as clearly beneficial. One of these occurred in the framework region and the second occurred in CDR L2. The latter was distinct from the beneficial mutations identified in the directed library (Figure 4).

#### 2.2.3 Summary of screening results

Considering the 11 mutations that clearly improved binding no general pattern is present but rather various types of substitutions are represented. Three mutations were to amino acids of the same class as the amino acid in the parental sequence. Three mutations were from uncharged amino acids to arginine. Two mutations were from polar, uncharged S to hydrophobic amino acids (H and Y). Finally, one mutation was from the positively charged amino acid K to the negatively charged amino acid E, and one mutation was from polar, uncharged S to proline.

### 2.3 Binding studies reveal enhanced binding affinity for 12 new F5‐derived Abs

**FIGURE 5:**
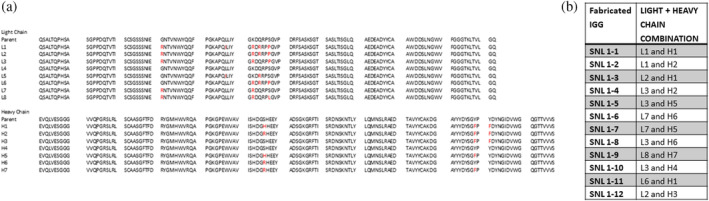
Sequences of 12 fabricated IgG variants of F5. (a) Sequence of parental and mutant light and heavy chains, with mutated residues highlighted in red. (b) Combinations of each mutated light and heavy chain sequence incorporated into 12 fabricated IgG variants.

**FIGURE 6:**
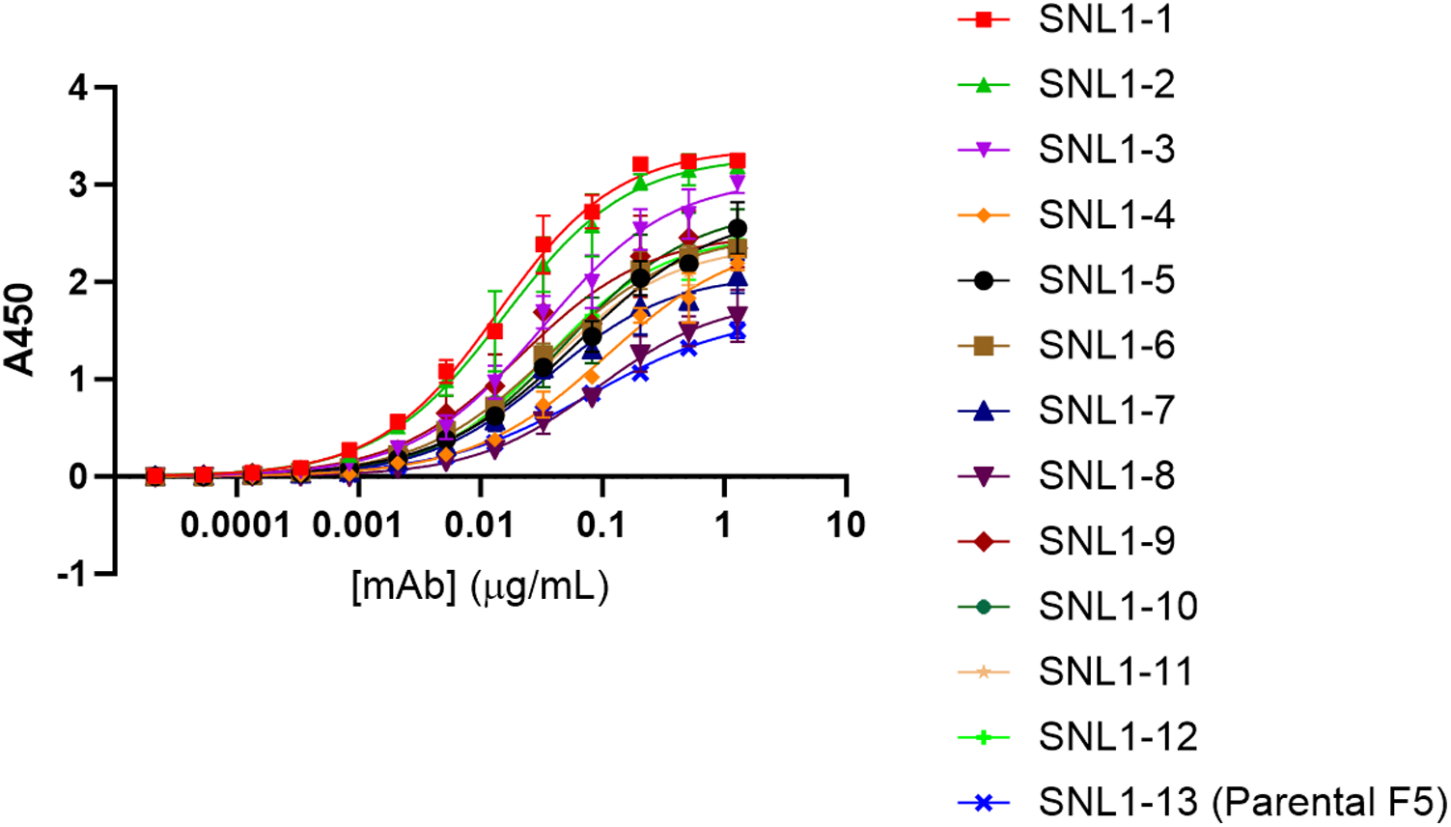
ELISA for IgGs binding to recombinant TC‐83 E1E2 heterodimer.

For construction of IgG variants, heavy and light chain sequences were designed to contain multiple combinations of mutations identified in our directed and random mutagenesis library screens (Figure S10). Individual mutant light and heavy chain sequences, as well as combinations of light and heavy chain sequences used to construct each fabricated IgG variant are listed in Figure 5. These IgGs were tested for binding to the recombinant TC‐83 E1E2 heterodimer by ELISA and by bilayer interferometry (BLI). The ELISA results, shown in Figure 6, indicate increased binding for all 12 of our mutant Abs (SNL1‐1 to SNL1‐12) relative to the parental F5 Ab (SNL1‐13). Of the 12 mutational combinations tested, some yielded only a very modest improvement on binding to TC‐83 (SNL1‐4 and SNL1‐8). Our top binding candidates SNL1‐1 and SNL1‐2, which included the most potent beneficial mutations, showed a substantial improvement in binding relative to the parental F5 (SNL1‐13).

**FIGURE 7:**
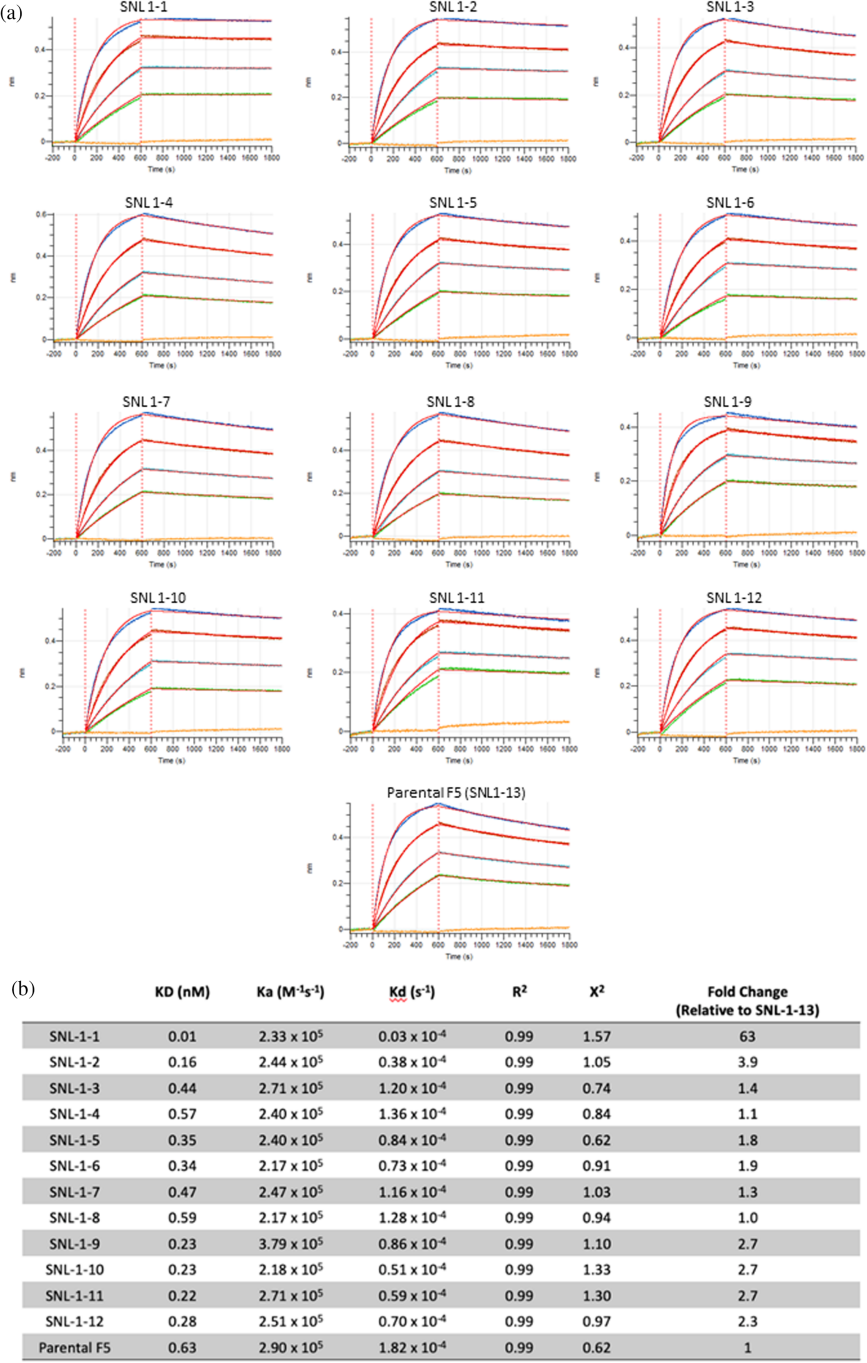
Bilayer interferometry for the IgGs binding to the TC‐83 E1E2 heterodimer. (a) Association/dissociation curves for each IgG, and (b) calculated KD, Ka, and Kd values based on 1:1 Global kinetic model.

BLI results for our designer Ab binding to recombinant E1E2, shown in Figure 7, agree well with the ELISA data and provide quantitative values for binding affinity. Of the mutant candidates tested, the largest improvement in binding relative to the parental occurred for SNL1‐1 and SNL1‐2, showing a 63 fold and 3.9 fold decrease in the equilibrium dissociation constant (KD) relative to F5 (SNL‐13) in our assay. When taking a closer look at the BLI binding data, we noticed that while there is some modest improvement in the Ka (association) for many of the mutants tested, all fell within a relatively close range (2.90 × 10^5^ to 2.17 × 10^5^ M^−1^ s^−1^), suggesting that the mutations made did not drastically affect the association rate of F5 with TC‐83 E1E2. Additionally, the top candidates SNL1‐1 and SNL1‐2 were not at the extreme end of this range with values of 2.33 × 10^5^ and 2.44 × 10^5^ M^−1^ s^−1^, respectively, suggesting that improvement in association was not the main driver of their selection. Interestingly, while all mutant candidates showed improvement in the Kd (off rate) relative to the parental F5, most mutants show modest improvement (≤4.8 fold decrease in Kd) while SNL1‐1 showed a drastic improvement in Kd (60.3 fold decrease). Interestingly, this suggests that the combination of binding enhancement mutations as well as the framework mutation included in SNL1‐1 result in a modest increase in association rate, but a significant improvement in dissociation rate, resulting in a more stable antigen–Ab interaction (Figure 7).

**FIGURE 8:**
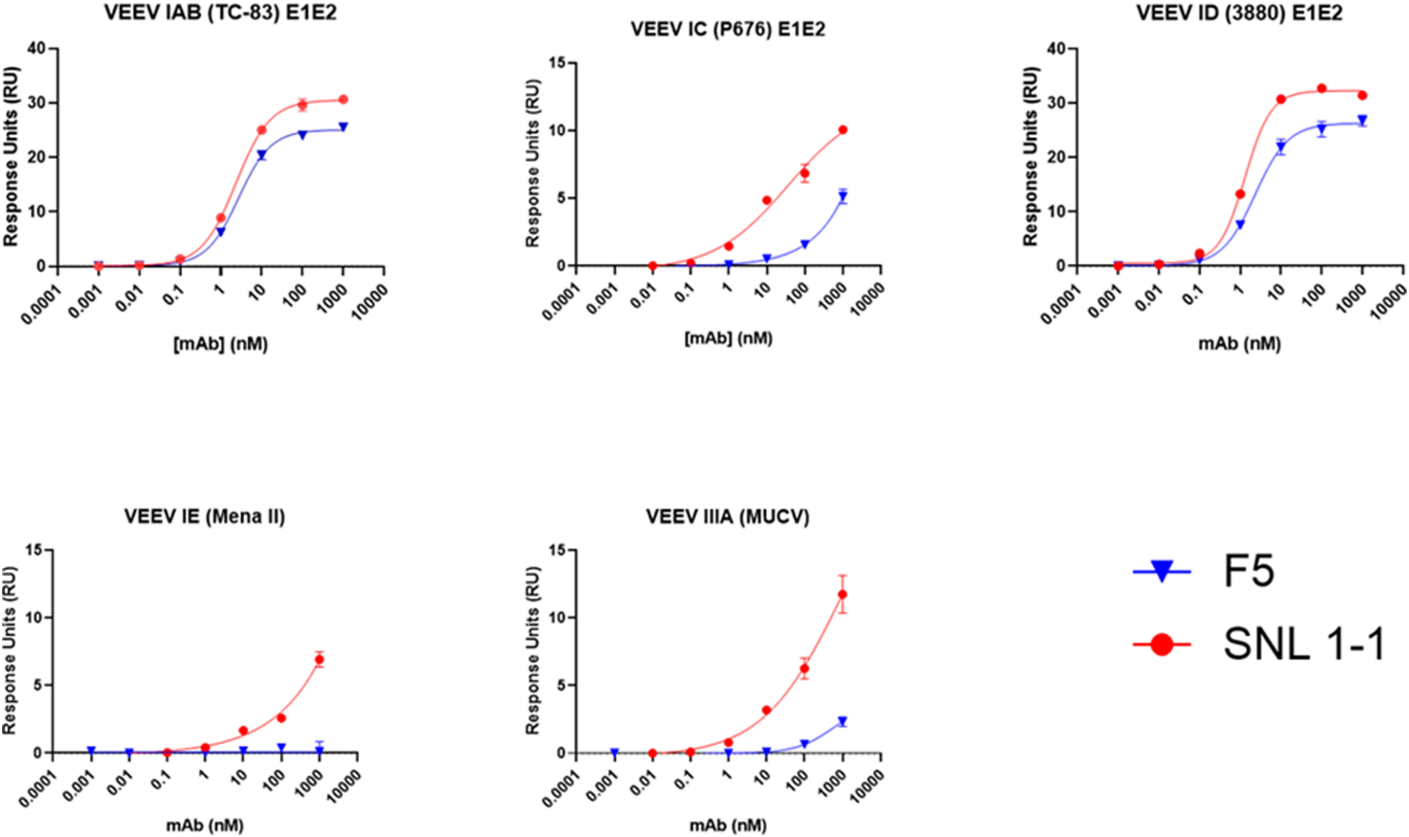
Gyros binding analysis of parental F5 and SNL1‐1 to E1/E2 heterodimer from related VEEV Subtypes. Binding screens conducted on Gyrolabs Xplore against purified E1E2 from VEEV subtypes IAB (TC‐83), IC (P676), ID (3880), IE (MenaII), and IIIA (MUCV). Fit curves generated using nonlinear least squares regression.

Interestingly, although not an explicit design goal, we also noticed that relative to the parental Mab, SNL1‐1 has increased breadth to closely related subtypes of VEEV. In our Gyrolabs Explore binding assay, which is optimized to detect improvements in weak antigen binding interactions, the parental F5 shows potent binding to E1E2 heterodimer from subtype IAB (TrD and TC‐83) and ID (3880), but significantly weaker binding to E1E2 from subtypes IC (P676), IE(MenaII), and IIIA (MUCV). In this assay, SNL1‐1 shows minor improvement in binding to both subtypes IAB and ID but SNL1‐1 shows substantial improvement against subtypes which F5 binds weakly, with the best improvements seen in binding to subtypes IC and IIIA (Figure 8).

### 2.4 Despite enhanced binding, SNL1‐1 does not improve efficacy against VEEV‐TC83

**FIGURE 9:**
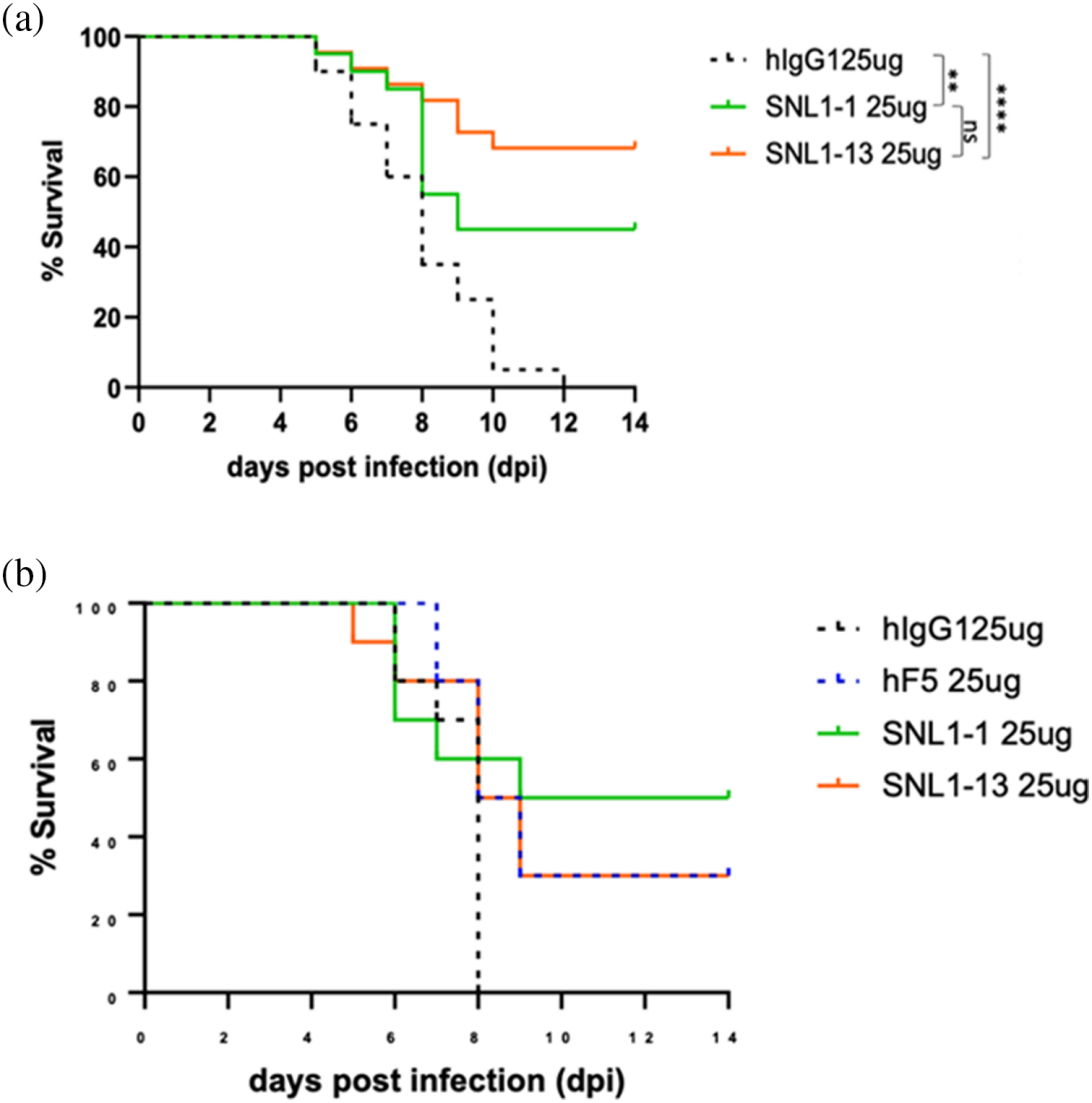
Parental and modified antibodies display similar therapeutic efficacy during lethal VEEV TC‐83 infection. Mice (*n* = 10) were infected intranasally with 5 × 10^7^ PFU VEEV TC‐83 and dosed with hF5, SNL1‐1, SNL1‐13, Abs1, Abs13, or isotype control antibodies at (a) +24 h and (b) +48 h post infection. Morbidity and mortality were assessed daily. **p < 0.01, ****p < 0.0001.

To determine the impact of enhanced Ab‐binding to recombinant E1E2 on the capacity to neutralize live virus, we performed a plaque reduction neutralization test (PRNT) with our mutant Abs. PRNT_50_ values are listed in Table 1. The raw data for percent neutralization versus Ab concentration are given in Figure S11. The results show no statistical improvement in neutralization for all 12 designed antibodies over the parental. Despite the lack of improved neutralization capacity, non‐neutralizing Abs have shown efficacy against VEEV demonstrating that neutralization potency is not the sole driver of protective effect. We tested the ability of our top candidate, SNL1‐1, which displayed the highest binding affinity for E1E2, to protect against infection with VEEV‐TC83 *in vivo* using an established mouse model of lethal infection (PMID 18313150). Since F5 has previously been reported to be up to 100% effective at preventing lethal infection when delivered prophylactically (Hunt et al. 2010), we assessed efficacy of SNL1‐1 when delivered therapeutically at +24 and +48hpi. The results of the survival experiments are shown in Figure 9. Treatment with both SNL1‐1 and the parental F5 (SNL1‐13) resulted in significant protection against infection when delivered at +24hpi (Figure 9a). Protection against infection was also seen at +48hpi (Figure 9b), although increased survival was not statistically significant at this timepoint. There was no statistical significance in survival observed between SNL1‐1 and F5 treated groups at either time point.

**TABLE 1:** Plaque reduction neutralization test data for IgGs.

Antibody	PRNT_50_ (μg/mL)
SNL1‐1	0.0009
SNL1‐2	0.0014
SNL1‐3	0.0010
SNL1‐4	0.0020
SNL1‐5	0.0015
SNL1‐6	0.0018
SNL1‐7	0.0015
SNL1‐8	0.0020
SNL1‐9	0.0041
SNL1‐10	0.0009
SNL1‐11	0.0016
SNL1‐12	0.0016
SNL1‐13 (parental)	0.0010

## 3 DISCUSSION

The uniqueness of this work is that our approach combines computational modeling with library screening to gain benefits from both approaches. Computational modeling was used to predict a set of potentially advantageous mutations. Library screening was then performed for nearly all combinations of a set of the predicted mutations. Computational modeling alone has the disadvantage that a single inaccurate mutational prediction can counteract the benefit of many accurate predictions. For random screening, the mutational sequence space is so large that a random search is very inefficient. Our approach makes use of the predictions of the computational modeling, and screening a library of nearly all combinations of the predicted mutations mitigates against the likelihood that some of the predictions will be incorrect. While others have shown that use of non‐natural amino acids can improve binding affinity of nanobodies to an antigen (Padhi et al. 2021), the present work involved only the 20 natural amino acids. While including non‐natural amino acids could improve this approach, engineered tRNAs for each non‐natural amino acid would be required.

We note that affinity is not the only characteristic predictive of therapeutic performance for mAbs or other biologic therapeutics or therapeutic platforms. Although minimal for a human mAb, there is a possibility that mutations in the CDR or framework regions alter the immunogenicity or stability profiles of a candidate therapeutic, each of which ultimately may alter therapeutic effect or pharmacokinetic profile. Consideration of these features of the candidate mAb F5 were out of scope for our study, however these are important features to consider for future biologic re‐design campaigns.

The combination of in silico analysis with directed library screening led to an improvement in the equilibrium dissociation constant (KD) of binding to recombinant E1E2 heterodimer by a factor of 63 for our top candidate SNL1‐1. This was the result of a modest improvement in the Ka (association) relative to the parental, but a significant improvement in the Kd (dissociation). This suggests that as opposed to improving overall affinity of F5, the mutations in SNL1‐1 primarily improved binding through stabilizing the F5‐E1E2 complex once formed. Although it is difficult to say definitively, we speculate that this is the product of attempting to improve overall affinity for a parental Ab which already has a KD in the sub‐nanomolar range for the target antigen. For this Ab–antigen interaction, the Kd (off‐rate) may be the primary space available for improvement.

Library testing of nearly all possible combinations of the specified mutations was critical as it enabled determination of the value of each mutation separately as well as the value of all combinations. Combining all predicted mutations into a single construct did not result in a successful clone. For the clones that had improved binding to the target, the mean number of mutations was 3.8. We expect that the predictive power will improve with better structural data and more accurate modeling. The directed library clearly outperformed the random library, identifying nine advantageous mutations versus two advantageous mutations for the random library. One of the beneficial mutations in the random library was in the framework region which was not addressed in the directed library. Sequence Tolerance and dTERMen contributed roughly equally in terms of successful predictions. FlexddG, employed after the experimental screening, correctly identified all the beneficial mutations as favorable. However, only four of the experimentally‐determined favorable mutations were predicted to be highly favorable by FlexddG. Had FlexddG been performed prior to library generation, most likely only those four would have been selected for inclusion in the library. We conclude that for this case Sequence Tolerance and FlexddG were of comparable value for predicting favorable mutations. Sequence Tolerance is substantially less computationally intensive than FlexddG and is available on the ROSIE public server.

The successful prediction of mutations to improve binding despite the absence of high‐resolution structural data for the H3 loop may be partly explained by the fact that the large H3 loop likely inserts into the cavity in the center of the E1E2 trimer. In that case the computational modeling may simply have needed to successfully identify H3 residues likely to be in contact with the antigen.

Ultimately, the mutations that were made to SNL1‐1 which showed significant improvement in binding to purified TC‐83 E1E2 antigen did not improve Ab functionality either by neutralization potency (Figure S10 and Table 1), or therapeutic dose after lethal challenge (Figure 9). Although we did not perform an extensive analysis of therapeutic dose or administration window, it is worth noting, that the parental F5 Ab has a sub‐nanomolar binding affinity, a PRNT50 value of 1 ng/mL, and provides 90%–100% protection when given pre‐exposure (−24 h) and 70%–90% protection when dosed +24 to +48 h post infection (hpi) in a lethal VEEV‐TC83 model (Hunt et al. 2010; Schwedler et al. 2024). This likely suggests that the native binding activity of the parental F5 Ab is sufficient for potent neutralization and protection, and the further increase in antigenic binding has diminishing returns for therapeutic potential and does not improve the post‐exposure therapeutic profile. We have recently demonstrated that Fc effector function contributes to the protective effect of F5 administered 48 h post infection (Schwedler et al. 2024), so it is possible that a combination of the affinity enhancing mutations made here, paired with additional mutations enhancing Fc effector function may improve therapeutic potency during this critical post‐exposure window. Additionally, although we were not able to complete a thorough analysis of protective effect across the VEE serocomplex viruses, we did note that while the mutations in SNL1‐1 improved/maintained potent binding interactions to antigen from subtypes IAB and ID, it also improved reactivity against antigens from subtypes IC and IIIA suggesting the potential for a more cross‐protective therapeutic profile relative to the parental Ab.

Although we did not observe noticeable therapeutic improvement against the target strain to an already potently neutralizing Ab, we propose that a similar process could be used to improve the therapeutic effect of an Ab with a less favorable Ab–antigen interaction such as when re‐targeting an existing Ab against a new emerging viral variant.

## 4 CONCLUSIONS

Through a combination of computational modeling and experimental library screening we substantially improved the affinity of F5 for recombinant E1E2 of VEEV (TC‐83). This is especially significant because high‐resolution structural data were not available for either F5 or VEEV and the H3 loop of F5 was comprised of 20 residues which posed a severe challenge for structural modeling. However, while the approach led to improvement in binding to recombinant E1E2, the improvement did not translate to improved binding to the virus or to in vivo efficacy against VEEV. In addition to increased binding to E1E2 of TC‐83, SNL1‐1 also showed improved binding to other VEEV subtypes (IC, IE, IIIA), demonstrating the potential for re‐targeting existing therapeutic antibodies against new emerging pathogenic variants.

## 5 MATERIALS AND METHODS

### 5.1 Computational methods

We modeled the interaction of F5 with VEEV‐TC83 (subtype IAB) and F5 with subtypes IV, and V using the Rosetta Modelling Suite. Homology models for the surface E1E2 trimers of these viruses were generated from the VEEV‐TC83 model 3J0C (Zhang et al. 2011) along with cryoEM data for F5 bound to VEEV‐TC83 (EMD‐2645) (Porta et al. 2014). The H3 loop of F5, comprising 20 residues, exceeds the length for which current modeling approaches can determine a unique structure (Weitzner et al. 2017; Weitzner and Gray 2017). Nine structures were generated using RosettaAntibody, PIGS (Prediction of ImmunoGlobulin Structure), and SWISS‐Model, all available in the public domain. Eight of the nine structures contained a kink in the H3 loop, since >80% of known structures of CDR H3 loops contain a C‐terminal kink (Weitzner and Gray 2017). These base Ab structures were docked against antigens of subtypes VEEV‐TC83, IAB, IV, and V using Rosetta protocols Docking2 and Snugdock on the ROSIE server available in the public domain (Lyskov et al. 2013). While a high‐resolution structure for F5/VEEV is not available, the published cryoEM structure indicates that F5 binds near the center of the three E2 monomers in the E1E2 trimer (Porta et al. 2014). Therefore, in the docking trials F5 was initially placed roughly 5 Å above the center of the trimer and models in which F5 bound to the trimer at locations away from the center were discarded. For each Ab/Ag pair one round of rigid docking (Docking2) was followed by two rounds of flexible docking (Snugdock). For the latter, the fast protocol was performed to avoid extensive remodeling of the H3 loop. I_sc is a docking score for the interface region where lower values indicate stronger binding and lower energy. In a docking trial the binding affinity was determined by the lowest value of I_sc (see Figure S2). The relative binding affinities for a particular F5 model structure with the different VEEV subtypes were then given by the relative values of the lowest I_sc for each Ab/Ag pair.

For predicting mutations to improve binding Rosetta‐based approaches and an informatics approach were employed. For the former, both Sequence Tolerance (Smith and Kortemme 2010; Smith and Kortemme 2011) and FlexddG (Barlow et al. 2018) developed by the Kortemme lab were used. Sequence Tolerance is an algorithm to predict the set of tolerated sequences for proteins and protein interfaces. From a single protein structure, a conformational ensemble is generated using Monte Carlo simulations involving backbone “backrub” and side chain moves. For each structure in the ensemble non‐native sequences are generated by incorporating each of the 20 amino acids at sites defined by the user. The non‐native sequences are evaluated for both interface binding and fold stability and compared to the native sequence, and sequences with scores near to or better than the native sequence are saved as being “tolerated.” This is performed for the entire conformational ensemble and the frequency with which each amino acid is included within the set of tolerated sequences is reported. Sequence Tolerance was performed using the ROSIE public server.

FlexddG is a method for modeling changes in interfacial binding free energies upon mutation within the Rosetta Protein Modelling Suite (Barlow et al. 2018). It applies Rosetta's interface ΔΔ*G* module, ddg‐monomer, to an ensemble of all‐atom configurations to account for conformational flexibility. The configurational ensemble is generated using Rosetta's “backrub” protocol that samples local side chain and backbone fluctuations for both partners of the protein–protein interface. In each instance, FlexddG evaluates ΔΔ*G* for a single inserted mutation at a single specified site. CDR lengths were held constant and FlexddG was used to determine ΔΔ*G* values for all 20 amino acids at each site targeted for mutation using recommended parameters of 35 structures, 5000 minimization iterations, and 35,000 backrub trials. For scoring, the generalized additive model (GAM) was used as described in Barlow et al. (2018). The scores that result from FlexddG are in Rosetta Energy Units (REUs) using the Rosetta Talaris energy function. The python script for FlexddG analysis was downloaded from https://github.com/Kortemme-Lab/flex_ddG_tutorial and was employed with the Rosetta Software Suite (2018.48.60516). The Rosetta Relax function was also used in the modeling procedure. FlexddG was performed using high‐performance computational resources at Sandia National Laboratories (SNL).

At the point in time at which the experimental library was designed and fabricated, we did not have the capability to perform FlexddG analysis and so the mutations selected for inclusion in the library were generated using Sequence Tolerance for F5 sites that were in contact with the antigen in the model structure. However, after the library screening we employed FlexddG analysis for the same sites to compare predictions between Sequence Tolerance and FlexddG.

dTERMen (Zhou et al. 2020) was also used to redesign all residues on the F5 Ab that contacted E1E2 in the structure selected for redesign. Briefly, given a template structure for design, dTERMen systematically breaks it down into constituent tertiary structural motifs (TERMs), then searches for low‐RMSD matches for each TERM from a representative database made from the Protein Data Bank (PDB) to gather an ensemble of similar structures. Amino‐acid sequence statistics in the resulting ensembles are then used to infer a second‐order model of sequence space compatible with the template structure (i.e., a pseudo‐energy model comprising self‐energies for each possible amino acid at each design position and pair energies for all possible amino acid pairs in interacting positions). Following the computation of self and pairwise pseudo‐energies for these positions (the pseudo‐energy table), linear programming‐based optimization was used to find the sequence S_min that minimized the total pseudo‐energy. Positions at which this optimal sequence differed from the Ab's original sequence were taken as the first set of mutations considered (Kingsford et al. 2005; Zhou et al. 2020).

In addition, to search for mutations that may be specific to the antigen at hand, additional mutations were generated by optimizing the total pseudo‐energy under a gentle “specificity gap.” A specificity‐gap captures the difference between the energy of an amino acid at a given design position in the context of the surrounding residues (in this case nearby antigen residues), and the energy of that amino acid in similar backbone structures but other sequence contexts. Thus, it can be used to bias mutations at each design position for the sequence context of the antigen versus other sequence contexts. It is computed in a pseudo‐energy table, wherein a specificity gap for amino acid a at position i is

$$G_{i}\left(a\right)=\sum_{k}\left[E_{i,k}\left(a,A_{k}\right)-\sum_{\beta \neq A_{k}}E_{i,k}\left(a,\beta \right)∙\frac{e^{-E_{i,k}\left(a,\beta \right)}}{\sum_{b\neq A_{k}}e^{-E_{i,k}\left(a,b\right)}}\right],$$

where $A_{k}$ is the amino acid at the antigen position k and $E_{i,k}\left(a,x\right)$ is the pair pseudo‐energy between amino acid a at design position i and amino acid x at antigen position k. A second set of mutations were then designed as described above, except with the additional constraint that the total specificity gap of the designed position had to be below 8 units. This procedure resulted in a set of mutations that was larger than could be incorporated into the experimental library. To downselect a subset of mutations for inclusion in the experimental library from among the set of predicted mutations, input complexes were repacked with PyRosetta (Chaudhury et al. 2010) using both sets of mutations, and then visually examined in PyMOL to manually screen for the most promising mutations based on shape complementarity and lack of clashing.

Subsets of mutations from the physics‐based approach (Sequence Tolerance) and the informatics‐based approach (dTERMen) were selected for inclusion in the experimental library.

### 5.2 Experimental methods

#### 5.2.1 DNA manipulation

pMTBip‐TC83‐E1E2 was constructed as follows and was adapted from other work to express recombinant E1‐E2 heterodimer from Sindbis virus (Li et al. 2010). A double‐stranded DNA fragment was commercially obtained (IDT) encoding the following TC83 structural protein coding sequences, E3 (1–59), E2 (1–344), and E1 (1–384). A Strep‐tag II sequence (GGGSWSHPQFEKGGGG) was present in between the coding sequence for E2 and E1 in addition to a C‐terminal hexa‐histidine tag. To produce soluble protein, trans‐membrane regions of E2 and E1 were not included. This DNA fragment was assembled with agarose purified pMT/Bip/V5‐HisA vector (Thermo), restriction digested with *BglII* and *XbaI*, and treated with phosphatase rSAP (NEB) using NEBuilder HiFi DNA master mix (NEB).

For Avi™‐tagged VEEV E1E2 subtypes, a linker sequence and Avi™ tag (SSGGLNDIFEAQKIEWHE) were inserted after the hexa‐histidine sequence using primer overlap PCR site directed mutagenesis. A novel XhoI restriction site was introduced within the linker sequence.

As above, double‐stranded DNA fragments were commercially obtained (IDT) encoding the structural protein coding sequences for E3, E2, Strep tag II, and E1 (aligning to regions used for IAB strain TC‐83) for VEEV subtype IC strain P676 (Accession # L04653), ID strain 3880 (Accession # L00930), IE strain MenaII (Accession # AF075252), and Mucambo virus (VEEV IIIA) (Accession # MF993533). These DNA fragments were assembled with agarose purified pMTBip‐TC83‐E1E2 His Avi™ vector described above, restriction digested with SpeI (unique within the E3 sequence insert) and *XhoI*, treated with phosphatase rSAP (NEB) using NEBuilder HiFi DNA master mix (NEB).

pSF‐CMV‐F5‐hIgG1 and pSF‐CMV‐F5‐λ were produced as follows. For the heavy and light chains of F5, double‐stranded DNA gBlocks (IDT) encoding the respective variable region was inserted upstream of the CH1‐CH3 region of hIgG1 and the lambda CL1. The genes were assembled with pSF‐CMV vector restriction digested with *NcoI* and *BamHI* using NEBuilder HiFi DNA master mix (NEB).

#### 5.2.2 Protein expression and purification

VEEV‐TC83 E1E2 glycoprotein was produced using a *Drosophila* S2 expression system. A stable S2 cell line which expressed VEEV‐TC83 E1E2 protein under an inducible promoter was generated as described in the technical literature provided by Gibco (Drosophila Schneider 2 (S2) Cells User Guide). In brief, S2 cells were cultured in complete Schneider's *Drosophila* Medium (10% heat‐inactivated FBS, 1% penicillin streptomycin). S2 cells were transfected using a CaCl_2_ protocol. Cells were transfected at 3.0 × 10^6^ cells/mL with 19 μg of pMTBip‐TC83‐E1E2 and 1 μg pCoPuro in a 35 mm plate as described in the manufacturers protocol for 18 h at 28°C. Cells were washed and media exchanged, then allowed to grow in the absence of selection agent for 48 h. Cells were then expanded in the presence of 7 μg/mL puromycin. Stable polyclonal S2 cells were expanded and adapted to EX‐CELL 420 serum‐free media (Sigma) supplemented with 0.1% Pluronic F‐68 over several passages with shaking. Protein expression was induced oncecell density reached 6 × 10^6^ cells/mL with 500 μM CuSO_4_.

Protein was allowed to express for 5 days after which the supernatant containing recombinant E1‐E2 protein was harvested. Cells were centrifuged at 4000*g* for 30 min at 4°C. Supernatant was then clarified by 0.45 μm filter prior to application to a 5 mL HisTrap Excel column (Cytiva) equilibrated with 20 mM Tris (pH 8.0), 300 mM NaCl. The column was washed extensively with buffer followed by washing with equilibration buffer with 40 mM imidazole. E1–E2 complex was eluted from the column with equilibration buffer supplemented with 500 mM imidazole. Fractions containing protein were dialyzed against 20 mM CHES (pH 9.5), 200 mM NaCl overnight at 4°C. Protein was concentrated and applied to Superdex 200 Increase 10/300 GL gel filtration column (Cytiva) equilibrated with 20 mM CHES (pH 9.5), 200 mM NaCl. Fractions containing E1–E2 complex were concentrated, aliquoted, and snap frozen with liquid N_2_. Protein was stored at −80°C.

#### 5.2.3 Library fabrication

To incorporate desired mutations at specific sites in the CDRs, codons were chosen using https://guinevere.otago.ac.nz/STATS/AA-Calculator.php. The oligos were designed to include as many of the prioritized predicted mutations as possible within a total diversity of ~10^8^, while avoiding stop codons and minimizing off‐target residues. 20–25 nt homology overlaps on both 3′ and 5′ ends were added for efficient annealing. The theoretical diversity of our library was 3.3 × 10^8^ based on the number of mutagenized positions and the number of incorporated amino acids at each position (Figure 2a,b). This was confirmed by sequencing (data not shown). During library construction a number of bacterial transformations were performed calculated to cover 10× theoretical diversity (each variant represented 10 times in the final library).

For generation and validation of the parental F5 scFv phagemid, the parental F5 scFv sequence was cloned into pAP‐III_6_ vector (Haidaris et al. 2001) in VH‐VL orientation with (GGGS)_4_ linker. This cloning event fused F5 scFv to the coat protein III of bacteriophage M13. The phagemid was transformed into NEB® Turbo chemically competent *Escherichia coli* strain and phage supernatants were prepared as described after cell growth and transduction with M13‐K07 helper phage (NEB) at multiplicity of infection (MOI) of 10. Binding of F5 phage supernatants to VEEV E1E2 heterodimer was determined by ELISA.

Randomly mutagenized scFv library was produced as described in prior work (Holland et al. 2013). Targeted mutagenized scFv library was produced using modified Kunkel mutagenesis method as described elsewhere (Kunkel 1985). Briefly, to produce ssDNA phagemid template for library generation, Eco29K I restriction sequences (CCGCGG) were introduced in each CDR of the F5 Ab sequence. The ssDNA template was produced in CJ236 strain (NEB) as previously described (Holland et al. 2013). Oligo mixture comprising a mix of wild type (WT) and mutagenized sequences for each CDR was annealed to the ssDNA template, followed by the extension and ligation reactions to generate dsDNA products. The newly generated mutagenized plasmids were transformed into AXM688 *E. coli* strain (Holland et al. 2013) expressing Eco29KI restriction nuclease for removal of all non‐recombinant products from the library. To validate the presence and proportion of each designed mutation, 48 single colonies were selected and F5 scFv variants were obtained by colony PCR and analyzed by Sanger sequencing.

To initiate the first round of screening, ELISA plates (Nunc Maxisorp) were coated with purified E1E2 VEEV‐TC83 heterodimer (100 μL, 10 μg/mL) overnight at 4°C. Phage library was added to the plates at approximately 1 × 10^11^ transforming units and incubated for 2 h at RT. The plates were then extensively washed and bound phage eluted and propagated in *E. coli* to be used for the next round of screening. The screen proceeded essentially as described previously (Batonick et al. 2016) through three rounds of selection with decreasing concentrations of the E1E2 heterodimer (3 μg/mL in round 2 and 1 μg/mL in round 3). After round 3, clonal phage was prepared from single colonies, and phage ELISA performed as described previously (Batonick et al. 2016) against E1E2 heterodimer coated on ELISA plates at 3 μg/mL (100 μL/well). Mutational analysis of the selected F5 variants was performed by PCR from the glycerol stock of single phage colonies followed by Sanger sequencing.

#### 5.2.4 Virus stocks

Vero cells (African green monkey kidney, ATCC CCL‐81) were cultured in minimum essential medium alpha (alpha MEM) supplemented with 10% fetal bovine serum (FBS), 100 units/mL penicillin, and 100 μg/mL streptomycin (Invitrogen, 15070063), at 37°C in 5% CO_2_. VEEV‐TC83 was obtained from the NIH Biodefense and Emerging Infections Research Resources Repository, NIAID, NIH (NR‐63) and amplified in supplemented alpha MEM. TC83 stocks were amplified in Vero cells infected at a MOI of 0.1, 40 h post infection virus was harvested and cellular debris removed by centrifugation 1000*g* for 5 min. Clarified supernatant containing TC83 was either snap frozen as a crude preparation with liquid nitrogen and stored at −80°C or further purified by density ultracentrifugation. In brief, crude preparation of virus was added on top of a cushion of 20% sucrose in PBS and centrifuged in a SW 40Ti rotor (Beckman) at 30,000 rpm for 1.5 h. Media and sucrose were then carefully aspirated and purified virus was reconstituted in PBS and stored at −80°C. Viral titers were determined by plaque assay.

#### 5.2.5 ELISA

Binding of IgG candidates from display screening was evaluated against both recombinant E1–E2 glycoprotein by immunosorbent assay. For recombinant E1–E2, glycoprotein was diluted in 100 mM NaCO_2_ (pH 8.3) 150 mM NaCl and immobilized on 384‐well immulon HBX plates (Thermo) overnight at 4°C. After washing with PBST, plates were blocked for 2 h at 25°C with Pierce (PBS) Protein Free blocking buffer. After removing block, serial dilutions of IgG candidates were prepared in blocking solution and allowed to incubate for at least 2 h in the plate with gentle shaking. The plate was washed and 1:15,000 HRP‐conjugated goal anti‐human (Thermo) was added. Plates were developed with TMB Ultra (Thermo) and the reaction stopped by addition of equal volume 2M H_2_SO_4_. Absorbance was read at 450 nm.

#### 5.2.6 PRNT assays

Neutralization assays to determine the plaque reduction neutralization for candidate IgG antibodies were performed as follows. Serial dilutions of each Ab in alpha MEM were mixed with equal volume of 25 PFU TC83 virus to form Ab–virus complexes. This mixture was preincubated at 37°C with 5% CO_2_ for 1 h prior to addition of Vero E6 cells at ~80% confluency in 12‐well plates and allowed to incubate for 30 min. Overlays of 1.5 mL 0.5% agarose in MEM were added to each well and allowed to solidify. After 36–40 h plates were rinsed with PBS and stained and fixed with 0.25% crystal violet (w/v) and 0.2% paraformaldehyde (v/v) for 30 min prior to extensive washing with water. Plaques were counted and EC50 values were determined using GraphPad software using the following equation: Y = Bottom + (Top‐Bottom)/(1 + (IC50/X)^HillSlope^).

#### 5.2.7 Biolayer interferometry

Kinetic parameters for each anti‐VEEV IgG Ab were determined using an Octet® RED384 (Sartorius). Each IgG was immobilized on anti‐human Fc sensors at a concentration of 0.31 μg/mL in 10 mM phosphate (pH 7.4), 300 mM NaCl, 1 mg/mL BSA, 0.02% NP‐40. VEEV TC83 E1E2 was used as the analyte at concentrations of 25, 12.5, 6.25, and 3.125 nM, and sensograms were fit to 1:1 global kinetics.

#### 5.2.8 Gyrolabs explore immunoassay

Avi™‐tagged proteins were site‐specifically biotinylated with the BirA biotin‐protein ligase lyophilized reaction kit (Avidity). Immunoassays were performed on the Gyrolab xPlore™ (Gyrolab Protein Technologies) using the Bioaffy 1000 HC CD platform. In brief, biotinylated antigen was loaded at a concentration 1 μM, 10 fold dilutions series Ab concentration curves from a starting concentration of 1 μM were loaded as sample, columns were washed 3× with PBS‐T, and Goat Anti‐human F(ab′)2‐Alexa 647 (Jackson ImmunoResearch 109‐606‐003) was loaded at a concentration of 10 nM, and applied to all samples. All samples were applied in duplicate, and results shown are representative of at least two independent experiments.

#### 5.2.9 Animal studies

All animal work was approved by the Lawrence Livermore National Laboratory Institutional Animal Care and Use Committee. All animals were housed in an Association for Assessment and Accreditation of Laboratory Animal Care (AAALAC)‐accredited facility. C3H/HeN female mice (Charles River) ranging in age from 4 to 8 weeks were inoculated intranasally with 5 × 10^7^ pfu VEEV‐TC83 under anesthesia (4%–5% isoflurane in oxygen). Animals were dosed with therapeutic antibodies at +24 or +48 h post infection via intraperitoneal injection (4 mg/kg in PBS). Mice were monitored daily for signs of morbidity up to 14 days post infection (dpi). Animals were humanely euthanized by CO_2_ asphyxiation upon signs of severe disease. Survival data were analyzed for significance using the Log‐rank (Mantel‐Cox) test using Prism software (GraphPad, La Jolla, CA).

## AUTHOR CONTRIBUTIONS

**Christopher A. Sumner:** Investigation; writing – original draft; writing – review and editing; formal analysis; visualization. **Jennifer L. Schwedler:** Investigation; writing – review and editing; formal analysis; writing – original draft. **Katherine Maia McCoy:** Investigation. **Jack Holland:** Investigation. **Valerie Duva:** Investigation. **Daniel Gelperin:** Investigation. **Valeria Busygina:** Investigation. **Maxwell A. Stefan:** Investigation; writing – original draft; formal analysis. **Daniella V. Martinez:** Investigation. **Miranda A. Juarros:** Investigation. **Ashlee M. Phillips:** Investigation. **Dina R. Weilhammer:** Supervision; investigation. **Gevorg Grigoryan:** Project administration; investigation; supervision. **Michael S. Kent:** Conceptualization; formal analysis; project administration; writing – original draft; investigation; visualization. **Brooke N. Harmon:** Conceptualization; project administration; funding acquisition.

## CONFLICT OF INTEREST STATEMENT

The authors declare no conflicts of interest.

## Supporting information


**Data S1.** Supporting Information.

## Data Availability

The data that support the findings of this study are available from the corresponding author upon reasonable request.

## Footnotes

1 In retrospect, a better approach would have been to generate a very large number of models and analyze the models for convergence to a small set of structures.

2 The efficacy of this approach would certainly be improved if resources allow for mutational analysis to be performed for more than a single structure.
