# Supplementary material for: Combining computational modeling and experimental library screening to affinity‐mature VEEV‐neutralizing antibody F5
Source: Protein Sci. 2025 Jan 22;34(2):e70043. doi: 10.1002/pro.70043 (PMC11752144; doi:10.1002/pro.70043)
Supplement: Supplementary file 1 — Data S1. Supporting Information. [file PRO-34-e70043-s001.pdf]

Supporting information for

Combining computational modeling and experimental library screening to affinity-mature VEEV-neutralizing antibody F5

Christopher A. Sumner†, Jennifer L. Schwedler†, Katherine M. McCoy¶, Jack Holland♯, Valerie Duva§, Daniel Gelperin§, Valeria Busygina§, Maxwell A. Stefan†, Daniella V. Martinez Ŧ, Miranda A. Juarros Ŧ, Ashlee M. Phillips‡, Dina R. Weilhammer‡, Gevorg Grigoryan¶ ♯ Michael S. Kent Ŧ *, Brooke N. Harmon†*

† Biotechnology and Bioengineering Department, Sandia National Laboratories, Livermore, CA

‡ Biosciences and Biotechnology Division, Lawrence Livermore National Laboratories, Livermore, CA

Ŧ Molecular and Microbiology Department, Sandia National Laboratories, Albuquerque, NM.

¶ Dept. of Molecular and Cell Biology, Dartmouth College

♯ Dept. of Computer Science, Dartmouth College

§ Abcam, 688 East Main Street, Branford, CT 06405

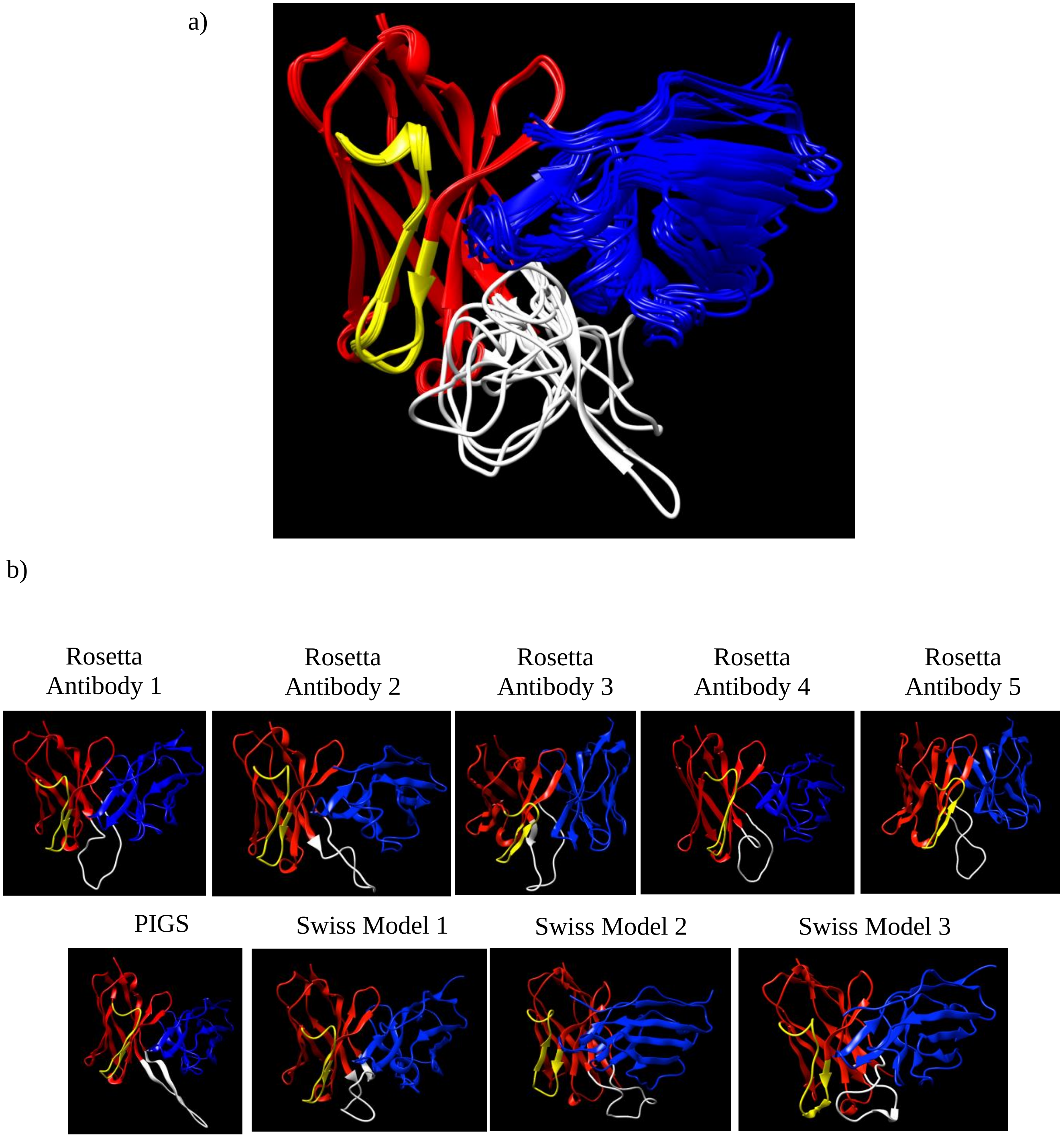

Figure S1. Structures of the nine F5 models used in this work, shown overlapped in a) and displayed separately in b). The H2 loop of F5 (VISHDGSHEEYADSG) is shown in yellow and the H3 loop of F5 (DGAYYYDYSGYPYDYNGIDV) loop is shown in white. The H2 loop structure is nearly identical in eight of the nine structures, but the H3 loop varies greatly among the 9 models.

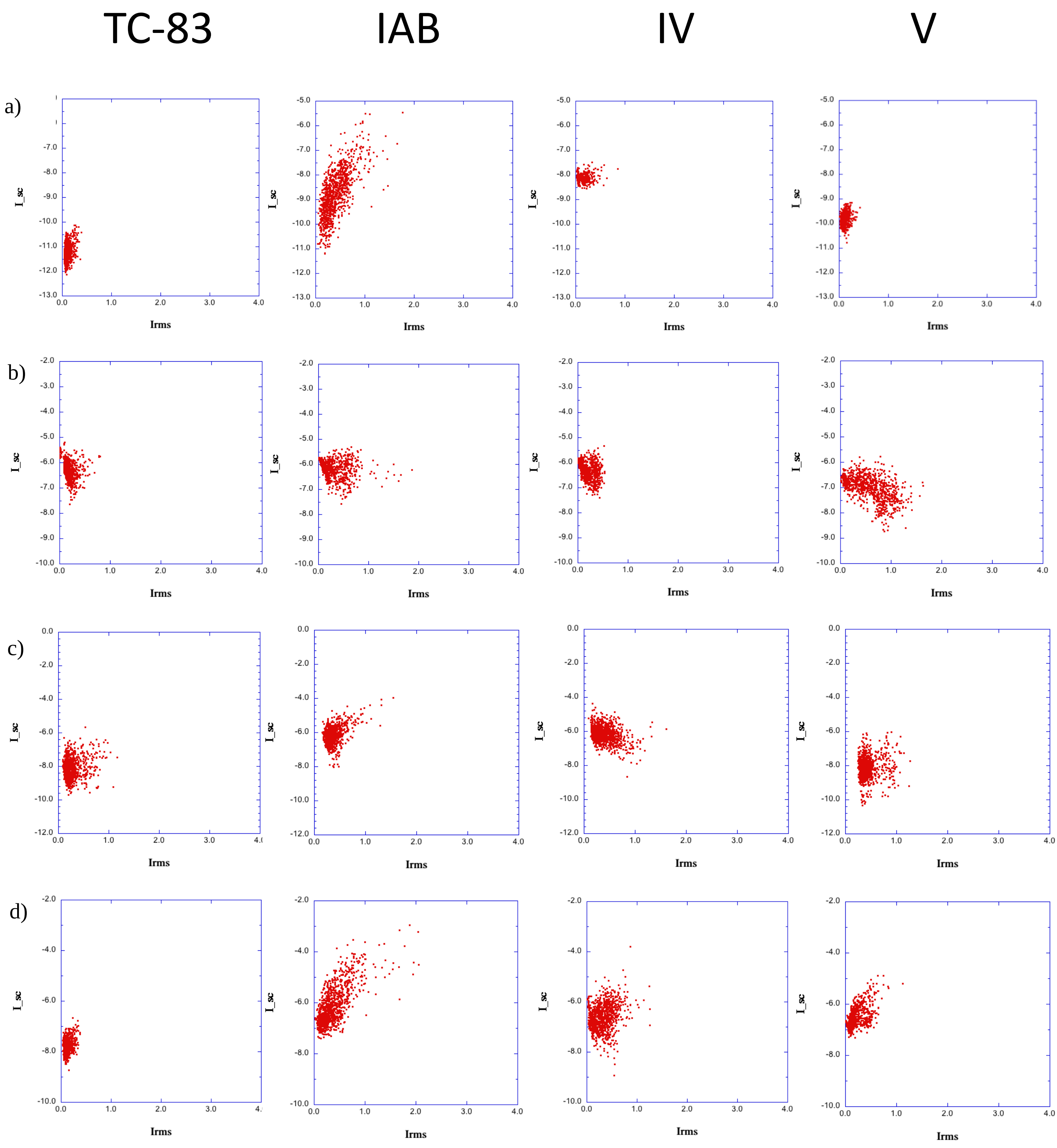
TC-83
IAB
IV
V
a)
b)
c)
d)
I_sc
Irms

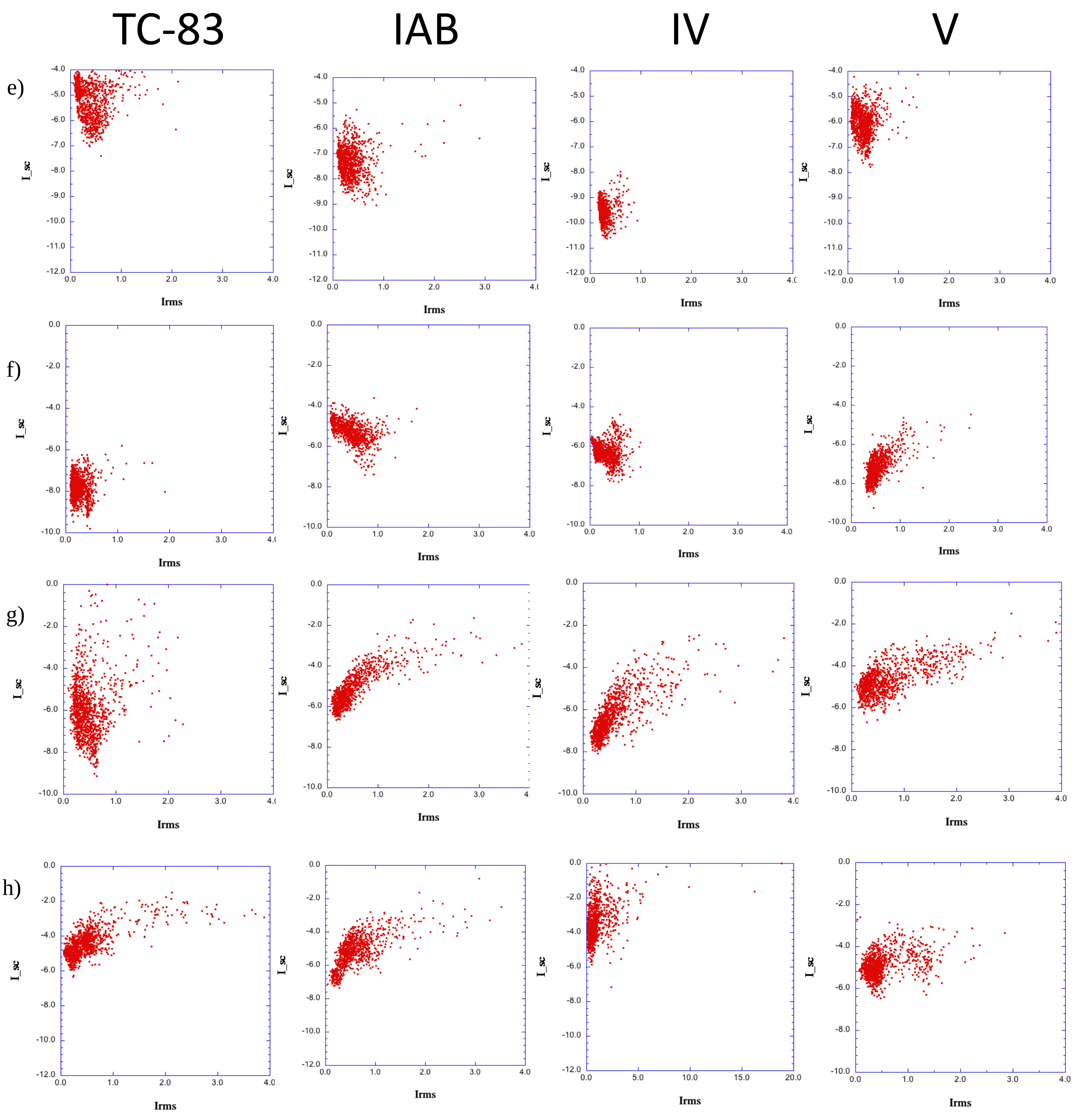
TC-83
IAB
IV
V
e)
f)
g)
h)
I_sc
Irms

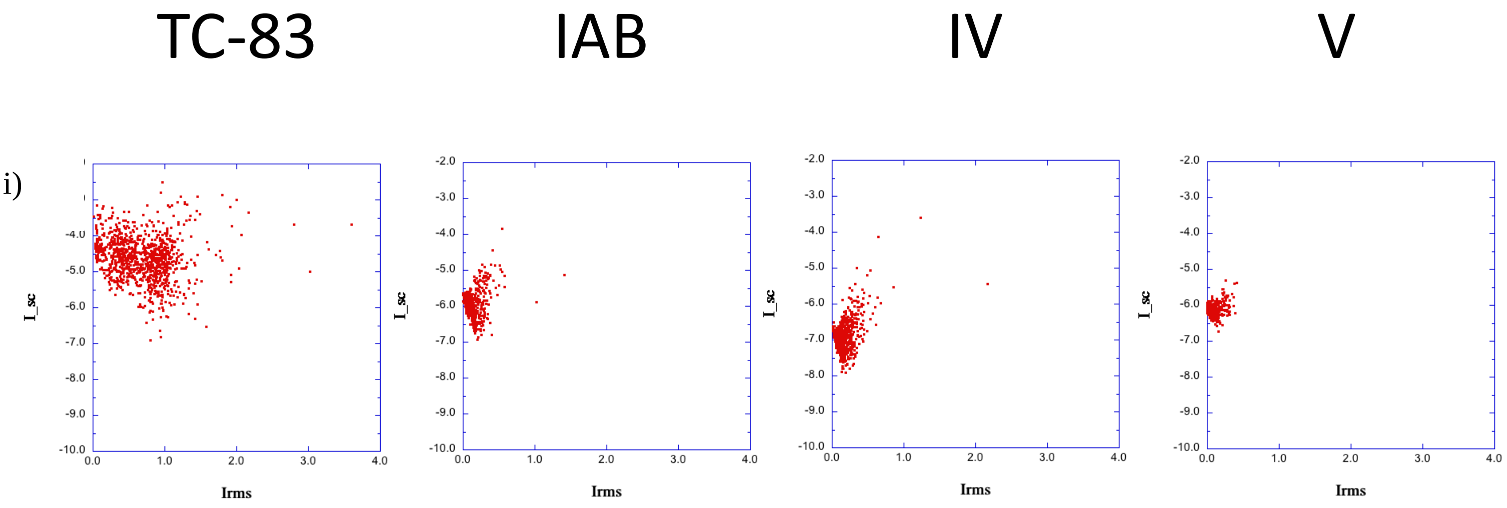

Figure S2. Docking scores for the following antibody structures: a) RosettaAntibody1, b) RosettaAntibody2, c) RosettaAntibody3, d) RosettaAntibody4, e) RosettaAntibody5, f) PIGS, g) SwissModel1, h) SwissModel2, i) SwissModel3. I_sc is a docking score for the interface region where lower values indicate stronger binding and lower energy. Irms is the root mean square deviation of C_α atoms from the initial configuration.

H1 Loop	native sequence	G	F	T	F	D	R	Y	G	M	H
	sequence tolerance predictions	G (90-100%)<br>S (0-10%)	M (30-40%)<br>T (20-30%)<br>D (0-10%)	D (20-30%)<br>Q (10-20%)<br>E (10-20%)<br>N (0-10%)	F (30-40%)<br>A (30-40%)<br>S (10-20%)<br>G (10-20<br>D (0-10%)	D (90-100%)<br>E (0-10%)	R (30-40%)<br>L (10-20%)<br>E (10-20%)<br>D (10-20<br>K (0-10%)	T (30-40%)<br>A (10-20%)<br>S (10-20%)<br>M (10-20%)<br>H (0-10%)	G (90-100%)<br>S (0-10%)	A (50-60%)<br>S (20-30%)<br>M (0-10%)	H (50-60%)<br>N (10-20%)<br>T (10-20%)<br>D(0-10%)

H2 Loop	native sequence	V	I	S	H	D	G	S	H	E	E	Y	A	D	S	G	K	G
	sequence tolerance predictions	V (80-90%)<br>T (0-10%)	I (80-90%)<br>V (10-20%)<br>T (0-10%)	S (90-100%)<br>D (0-10%)	H (90-100%)<br>N (0-10%)	D (80-90%)<br>S (0-10%)	G (90-100%)<br>N (0-10%)	R (90-100%)<br>K (0-10%)	R (50-60%)<br>K (10-20%)<br>Y (0-10%)	R (10-20%)<br>S (10-20%)<br>Q (10-20%)<br>A (10-20%)<br>M (10-20%)	S (50-60%)<br>D (40-50%)<br>A (0-10%)	Y (70-80%)<br>F (10-20%)<br>R (0-10%)	S (40-50%)<br>A (10-20%)<br>D (10-20%)<br>N (10-20%)<br>H (0-10%)					

H3 Loop	native sequence	D	G	A	Y	Y	Y	D	Y	S	G	Y	P	Y	D	Y	N	G	I	D
	sequence tolerance predictions	D (90-100%)<br>S (0-10%)	G (90-100%)<br>N (0-10%)	A (70-80%)<br>S (20-30%)<br>T (0-10%)	W (50-60%)<br>Y (20-30%)<br>F (10-20%)<br>H (0-10%)	F (50-60%)<br>Y (30-40%)<br>R (0-10%)	S (60-70%)<br>A (20-30%)<br>G (10-20%)<br>D (0-10%)	D (30-40%)<br>H (20-30%)<br>M (0-10%)	K (50-60%)<br>G (20-30%)<br>A (10-20%)<br>S (0-10%)	S (40-50%)<br>M (40-50%)<br>A (0-10%)	G (90-100%)<br>A (0-10%)	H (90-100%)<br>Y (0-10%)	E (40-50%)<br>D (20-30%)<br>Q (10-20%)<br>H (0-10%)	F (40-50%)<br>Y (30-40%)<br>E (10-20%)<br>D (0-10%)	G (90-100%)<br>S (0-10%)	W (80-90%)<br>F (10-20%)<br>Y (0-10%)	E (40-50%)<br>H (30-40%)<br>M (0-10%)	G (90-100%)<br>A (0-10%)	I (40-50%)<br>V (10-20%)<br>M (10-20%)<br>H (0-10%)	E (50-60%)<br>D (10-20%)<br>T (0-10%)

L1 Loop	native sequence	S	G	S	S	S	N	I	E	G	N	T	V	N	W
	sequence tolerance predictions		G (90-100%)<br>S (0-10%)	Q (10-20%)<br>S (10-20%)<br>T (10-20%)<br>L (0-10%)	S (20-30%)<br>T (20-30%)<br>K (10-20%)<br>A (10-20%)<br>R (0-10%)	S (20-30%)<br>N (10-20%)<br>D (10-20%)<br>Q (0-10%)	R (70-80%)<br>E (10-20%)<br>K (0-10%)	I (90-100%)<br>V (0-10%)	K (50-60%)<br>E (10-20%)<br>R (0-10%)	K (30-40%)<br>R (20-30%)<br>Q (0-10%)	S (80-90%)<br>N (10-20%)<br>A (0-10%)	M (30-40%)<br>T (10-20%)<br>V (10-20%)<br>N (10-20%)<br>I (10-20%)	V (80-90%)<br>T (0-10%)	D (20-30%)<br>T (20-30%)<br>A (20-30%)<br>S (10-20%)<br>N (10-20%)	W (90-100%)<br>F (0-10%)

L2 Loop	native sequence	Q	L	L	I	Y	G	K	D	Q	R	P	S	G	V	P	D
	sequence tolerance predictions		L (90-100%)<br>I (0-10%)	L (90-100%)<br>T (0-10%)	V (90-100%)<br>I (0-10%)	F (90-100%)<br>Y (0-10%)	H (90-100%)<br>G (0-10%)	R (80-90%)<br>I (10-20%)<br>D (0-10%)	D (50-70%)<br>N (0-10%)	E (80-90%)<br>Q (0-10%)	R (30-40%)<br>D (10-20%)<br>T (10-20%)<br>I (10-20%)<br>V (0-10%)	P (40-50%)<br>W (10-20%)<br>S (0-10%)	S (30-40%)<br>M (20-30%)<br>T (20-30%)<br>D (10-20%)<br>E (0-10%)	G (90-100%)<br>N (0-10%)	V (70-80%)<br>I (20-30%)<br>T (0-10%)	P (50-70%)<br>A (0-10%)	D (70-80%)<br>S (10-20%)<br>N (0-10%)

Figure S3. Results of Sequence Tolerance analysis of F5 residues in the CDR loops in the final refined F5/VEEV complex. The residues that contact the antigen are shown in pink. L3 does not contact the antigen in the modeled structure. The mutations chosen for inclusion in the experimental library are highlighted in yellow.

Chain	CDR loop	Original Residue	Location in F5/VEEV structure	Mutation predicted
H		E	1	T
H		V	2	P
H	H1	T	28	D
H	H1	D	30	S
H	H1	R	31	D
H	H2	H	52 A	P
H	H2	S	55	Y
H	H2	H	56	K
H		R	71	W
H		N	73	L
H		N	82 A	D
H		S	82 B	N
H	H3	K	94	I
H	H3	D	95	H
H	H3	G	96	R
H	H3	A	97	D
H	H3	Y	99	F
H	H3	Y	100	K
H	H3	D	100 A	K
H	H3	Y	100 B	D
H	H3	S	100 C	P
H	H3	Y	100 E	F
H	H3	Y	100 G	G
H	H3	D	100 H	N
H	H3	Y	100 I	V
H	H3	N	100 J	F
H	H3	I	100 L	F
H	H3	D	101	P
H	H3	V	102	Y
L	L1	E	30 A	F

L	L1	G	30 B	K
L	L1	N	31	G
L	L1	T	32	V
L	L2	I	48	L
L	L2	Y	49	D
L	L2	K	51	G
L	L2	Q	53	E
L	L2	R	54	I
L	L2	S	56	P
L	L2	D	60	P
L		A	64	G
L		K	66	A
L		S	67	E
L		W	91	P

Figure S4. Results of dTERMen analysis of F5 residues in the CDR loops in the final refined F5/VEEV complex using pseudo-energy alone. Differences from the predictions with specificity cutoff are highlighted in cyan.

Chain	CDR Loop	Original Residue	Location (in F5/VEEV structure)	Mutation Suggested
H		E	1	T
H		V	2	P
H	H1	F	27	E
H	H1	T	28	D
H	H1	D	30	S
H	H1	R	31	D
H	H1	Y	32	K
H	H2	H	52A	P
H	H2	S	55	Y
H	H2	H	56	K
H		R	71	W
H		N	73	L
H		N	82A	D
H		S	82B	N
H	H3	K	94	I
H	H3	D	95	H
H	H3	G	96	R
H	H3	A	97	D
H	H3	Y	99	R
H	H3	Y	100	K
H	H3	D	101	P
H	H3	V	102	Y
H	H3	D	100A	K
H	H3	Y	100B	D
H	H3	Y	100E	F
H	H3	Y	100G	G
H	H3	D	100H	N
H	H3	Y	100I	V
H	H3	N	100J	F
H	H3	I	100L	F
L	L1	E	30A	S
L	L1	G	30B	R
L	L1	N	31	H
L	L1	T	32	V
L	L2	I	48	L
L	L2	Y	49	D
L	L2	K	51	G
L	L2	Q	53	E
L	L2	R	54	I
L	L2	S	56	P
L		D	60	P
L		A	64	G
L		K	66	A
L		S	67	E
L		W	91	P

Figure S5. Results of dTERMen analysis of F5 residues in the CDR loops in the final refined F5/VEEV complex using energy with specificity cutoff (differences from predictions without specificity cutoff highlighted):

H1 Loop	native sequence	G	F	T	F	D	R	Y	G	M	H						
	predictions		E	D		S	D	K									
H2 Loop	native sequence	V	I	S	H	D	G	S	H	E	E	Y	A	D	S	G	K
	predictions				P			Y	K								
H3 Loop	native sequence	D	G	A	Y	Y	Y	D	Y	S	G	Y	P	Y	D	Y	N
	predictions			D		R	K	K	D	P		F		G	N	V	F
Li Loop	native sequence	S	G	S	S	S	N	I	E	G	N	T	V	N	W		
	predictions								F,S	K, R	G,H	V					
L2 Loop	native sequence	Q	L	L	I	Y	G	K	D	Q	R	P	S	G	V	P	D
	predictions				L	D		G		E	I		P				P

Figure S6. Mutations from dTERMen analysis of F5 residues in the CDR loops chosen for inclusion in the experimental library are highlighted in yellow. The residues that contact the antigen are shown in pink. L3 does not contact the antigen in the modeled structure.

		CDR L1			CDR L2			CDR H1	CDR H2		CDR H3			
	Clone rank	L31	L32	L33	L52	L54	L57	H32	H55	H56	H100C	H100E	H100G	H100H
		G	N	T	K	Q	S	Y	S	H	S	Y	Y	D
target AA		GKR	NSGH	TMV	KRG	QE	SMTP	YTK	SRY	HRK	SMP	YHF	YFG	DGN
possible AA		GKR E	NSGH DR	TMV A	KRG E	QE	SMTP L2	YKT2 NS2*	SRY CHP	HR5K2 NSQ2	SMP TL2	YHF L	YFG DCV	DGN S
Above Parental	1	R			R		P		H			F		
	2	R			R				R					
	3	R			R				H			F		
	4	R			R		P		R					
	5	R			R		L		R			F		
	6	R			R				H			F		
	7	R			R		P							
	8	R			E				R					
	9	R			R		P							
	10	R			E				Y				F	
	11	R			R		P		Y			F	F	
	12	R			R		P		R				F	
	13	R			R		P		R	N				
	14	R			R				Y					
	15	R			R		P		R				F	
	16				R				X	X		X		
	17	R												
	18	R			R		M		C				F	
	19	R			E				R					
	20	R			R				Y					
	21	R			R		P		H			F		
	22	R			R		P					F		
	23	R			E		T		R					
	24	R					T							
	25	R			R		L							
	26	R					T		Y				F	
	27	R			E									
	28				R		P		R			F		
	29	R			R		P		H					

Figure S7. Results of experimental library screening showing mutations in clones that had ELISA signal for binding to antigen that was greater than that of the parental sequence.

		CDR L1			CDR L2			CDR H1	CDR H2		CDR H3			
	Clone rank	L31	L32	L33	L52	L54	L57	H32	H55	H56	H100C	H100E	H100G	H100H
		G	N	T	K	Q	S	Y	S	H	S	Y	Y	D
target AA		GKR	NSGH	TMV	KRG	QE	SMTP	YTK	SRY	HRK	SMP	YHF	YFG	DGN
possible AA		GKR E	NSGH DR	TMV A	KRG E	QE	SMTP L2	YKT2 NS2*	SRY CHP	HR5K2 NSQ2	SMP TL2	YHF L	YFG DCV	DGN S
Similar to Parental	30	R			E		T						F	
	31		D	M	R			X		X		L	X	
	32	R					T		R				F	
	33	K			R		T							
	34	R			R		M	X						
	35	R			E		L		Y			F		
	36				R		M		H			F		
	37	R					R		C	N				
	38	R			R		T		C	S		F		
	39				E									
	40	K					T	T	R		P	L	V	N
	41	R			R		L		R				V	
	42	K	D	A			L	N	C					
	43				E		L		R			F		N
	44						T							

Figure S8. Results of experimental library screening showing mutations in clones that had ELISA signal for binding to antigen that was comparable to that of the parental sequence.

		CDR L1			CDR L2			CDR H1	CDR H2		CDR H3			
	Clone rank	L31	L32	L33	L52	L54	L57	H32	H55	H56	H100C	H100E	H100G	H100H
		G	N	T	K	Q	S	Y	S	H	S	Y	Y	D
target AA		GKR	NSGH	TMV	KRG	QE	SMTP	YTK	SRY	HRK	SMP	YHF	YFG	DGN
possible AA		GKR E	NSGH DR	TMV A	KRG E	QE	SMTP L2	YKT2 NS2*	SRY CHP	HR5K2 NSQ2	SMP TL2	YHF L	YFG DCV	DGN S
Less than Parental	45	E	G		E		T	X	C	S				
	46		H		G		M	T	C	Q			V	N
	47		G			E	T	T		S	P	F		N
	48	R	G	V			P	T	R			F		N
	49	R	H		R	E	T		R	N	T	F	V	N
	50	R	D	A		E	T					L	C	
	51	E	G				M	T	R	R	T	H	D	
	52	K	G				L			R	M	L		
	53	E	D				M	S	C	R	L	L	G	G
	54		S	M			P	N	R	N		H	C	S
	55	E	R		R		T	S	P	S				
	56		D	M		E	T	N	R	Q			D	
	57	K		M		E		T		N		L	D	N
	58		S			E	T	N	P		T	H	G	
	59	E	R	M	R			S	Y	Q	L	L	F	S
	60	K	D	A			L	T		R		H	D	S
	61		G	M			L	T	R	K	M	H	F	
	62		G	A		E	L	T		Q	L	L	V	
	63			V			P	T		K		L	C	
	64		D	V					H	K	P	H	V	N
	65	K	D				M	N	Y	K	T	L	D	N
	66	K		M		E	P	S	Y		P	L	C	
	67						M		R	R	L	L	V	N
	68	E	R	M	R		P	K	Y	N	M		F	S

Figure S9. Results of experimental library screening showing mutations in clones that had ELISA signal for binding to antigen that was lower than that of the parental sequence.

A.

position	L31	L47	L52	L54	L57
Parental	G	L	K	Q	S
mutant	R	I	R	R	P,L
L1	R	I	R	R	P
L2	R	L	R	R	P
L3	R	L	R	Q	P
L4	G	L	K	Q	S
L5	G	I	K	R	S
L6	G	L	R	R	P
L7	R	L	R	Q	S
L8	R	L	R	Q	L

B.

position	H55	H100E	H100G
Parental	S	Y	Y
mutant	R, H,Y	F	F
H1	H	F	F
H2	R	F	F
H3	S	F	F
H4	S	Y	Y
H5	H	F	Y
H6	R	Y	Y
H7	R	F	Y

Figure S10. Inclusion of beneficial mutations into fabricated IgGs. A) Light chain and B) Heavy chain beneficial mutations from library screens and composition of mutant chain sequences. Green – Native Residues, Orange – mutation from random library, White – mutation from directed library.

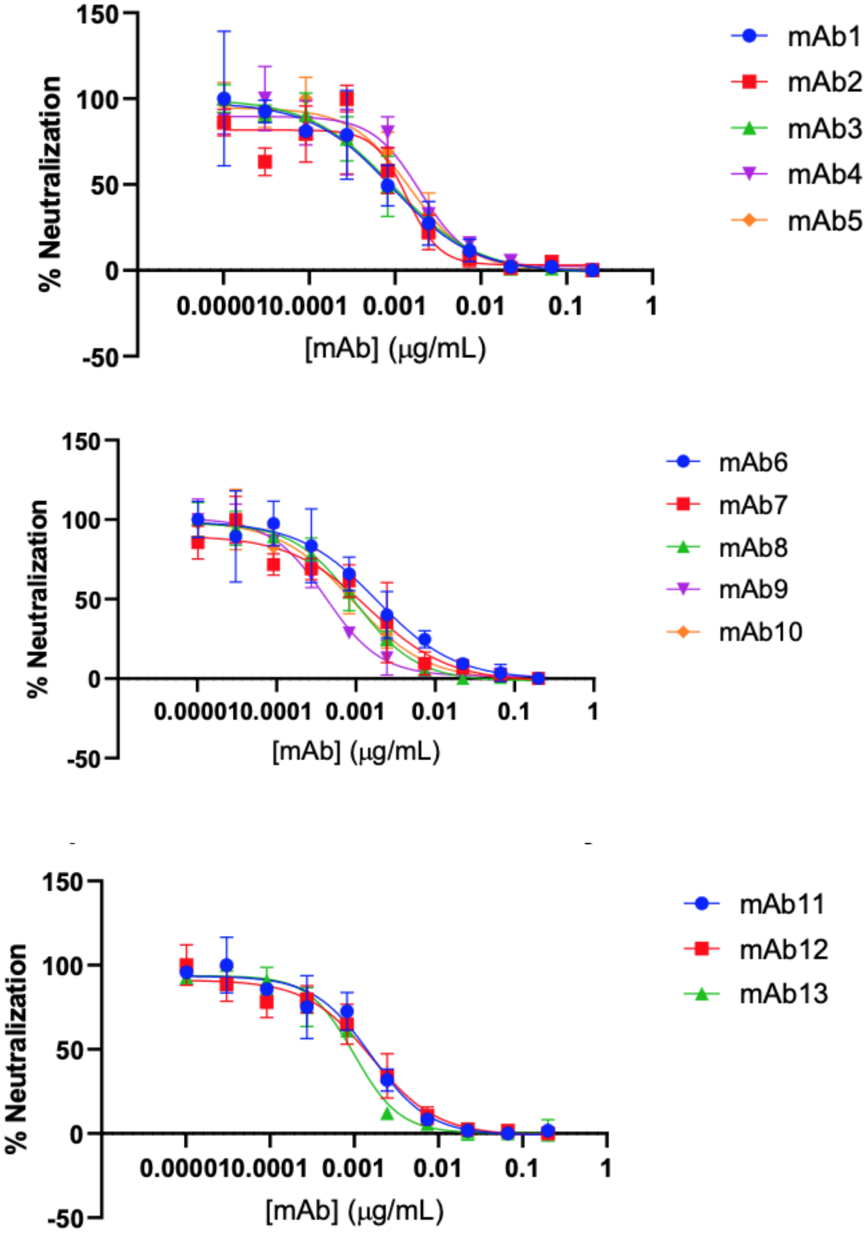

Figure S11. Raw data for Plaque Reduction Neutralization Test
